# Injectable hydrogel-based combination therapy for myocardial infarction: a systematic review and Meta-analysis of preclinical trials

**DOI:** 10.1186/s12872-024-03742-0

**Published:** 2024-02-21

**Authors:** Han Gao, Song Liu, Shanshan Qin, Jiali Yang, Tian Yue, Bengui Ye, Yue Tang, Jie Feng, Jun Hou, Dunzhu Danzeng

**Affiliations:** 1 School of Medicine, Tibet University, Lhasa, Tibet China; 2 School of Life Science and Engineering, Southwest Jiaotong University, Chengdu, Sichuan China; 3 West China School of Pharmacy, Sichuan University, Chengdu, Sichuan China; 4 School of Pharmacy, North Sichuan Medical College, Nanchong, Sichuan China; 5 School of Medicine, Southwest Jiaotong University, Chengdu, Sichuan China; 6 Department of Cardiology, Chengdu Third People’s Hospital, Chengdu, Sichuan China

**Keywords:** Hydrogel, Myocardial infarction, Combination therapy

## Abstract

**Introduction:**

This study evaluates the effectiveness of a combined regimen involving injectable hydrogels for the treatment of experimental myocardial infarction.

**Patient concerns:**

Myocardial infarction is an acute illness that negatively affects quality of life and increases mortality rates. Experimental models of myocardial infarction can aid in disease research by allowing for the development of therapies that effectively manage disease progression and promote tissue repair.

**Diagnosis:**

Experimental animal models of myocardial infarction were established using the ligation method on the anterior descending branch of the left coronary artery (LAD).

**Interventions:**

The efficacy of intracardiac injection of hydrogels, combined with cells, drugs, cytokines, extracellular vesicles, or nucleic acid therapies, was evaluated to assess the functional and morphological improvements in the post-infarction heart achieved through the combined hydrogel regimen.

**Outcomes:**

A literature review was conducted using PubMed, Web of Science, Scopus, and Cochrane databases. A total of 83 papers, including studies on 1332 experimental animals (rats, mice, rabbits, sheep, and pigs), were included in the meta-analysis based on the inclusion and exclusion criteria.

The overall effect size observed in the group receiving combined hydrogel therapy, compared to the group receiving hydrogel treatment alone, resulted in an ejection fraction (EF) improvement of 8.87% [95% confidence interval (CI): 7.53, 10.21] and a fractional shortening (FS) improvement of 6.31% [95% CI: 5.94, 6.67] in rat models, while in mice models, the improvements were 16.45% [95% CI: 11.29, 21.61] for EF and 5.68% [95% CI: 5.15, 6.22] for FS.

The most significant improvements in EF (rats: MD = 9.63% [95% CI: 4.02, 15.23]; mice: MD = 23.93% [95% CI: 17.52, 30.84]) and FS (rats: MD = 8.55% [95% CI: 2.54, 14.56]; mice: MD = 5.68% [95% CI: 5.15, 6.22]) were observed when extracellular vesicle therapy was used. Although there have been significant results in large animal experiments, the number of studies conducted in this area is limited.

**Conclusion:**

The present study demonstrates that combining hydrogel with other therapies effectively improves heart function and morphology. Further preclinical research using large animal models is necessary for additional study and validation.

**Graphical abstract:**

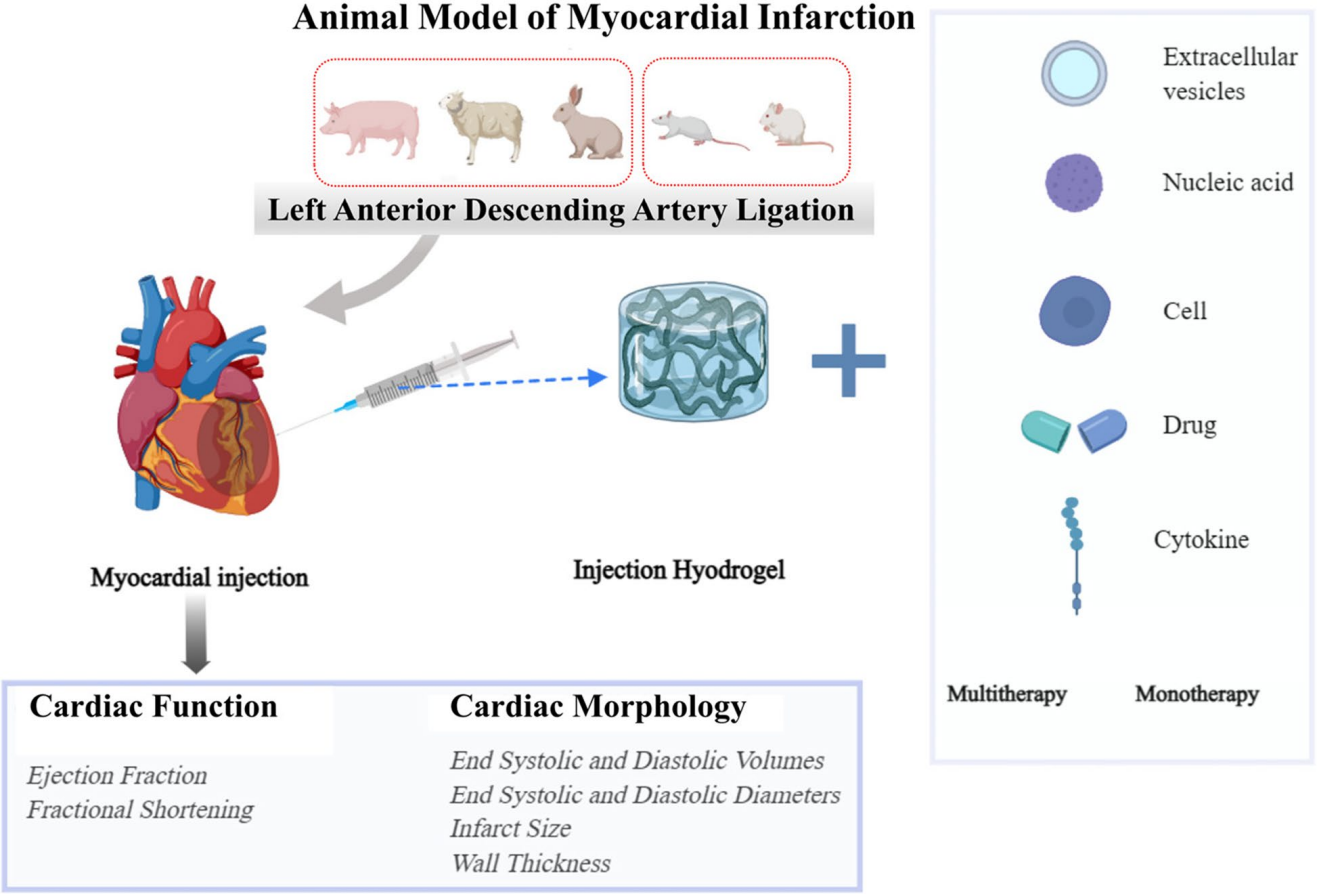

**Supplementary Information:**

The online version contains supplementary material available at 10.1186/s12872-024-03742-0.

## Introduction

Myocardial infarction, resulting from sudden ischemia and cell damage in the myocardial tissue, leads to irreversible cardiac impairment [1]. The recovery phase after injury involves both acute and chronic inflammation, which, coupled with increased cardiac load due to diminished heart function, exacerbates heart tissue damage. This detrimental cycle, known as “injury - increased cardiac load - heightened injury,” ultimately progresses to heart failure [2]. Although treatments for myocardial infarction include drug therapy, surgical device implantation, and organ transplantation, drug therapy is the most accessible option. Its goal is to decelerate the progression of cardiac injury by reducing the cardiac load. However, its effectiveness is limited and often accompanied by systemic toxicity and suboptimal drug utilization, which undermine the potential benefits of many clinical agents. Furthermore, myocardial infarction remains a significant cause of global morbidity and mortality [3].

Bioactive scaffolds, combined with bioactive drugs or cells to facilitate cellular attachments, have gained attention for their potential to promote tissue repair following myocardial infarction and reverse heart damage [4]. Currently, bioactive scaffolds take the form of hydrogels, patches, and nanoparticles [5]. Hydrogels, which are hydrophilic polymeric three-dimensional networks [6], possess suitable mechanical properties, moisturizing capabilities, biocompatibility, biodegradability, and biomimetic characteristics, all of which are crucial for sustained drug delivery and tissue regeneration [7]. Despite these advantages, hydrogels as biomaterials have a relative deficiency in bioactivity [8]. However, by incorporating various bioactive drugs, cells, and cellular appendages, hydrogels can exhibit anti-inflammatory, anti-apoptotic, and tissue repair capabilities. Targeted injections into the area of myocardial infarction can ensure the prolonged release of therapeutic agents, stabilizing therapeutic outcomes and improving prognosis [9].

Injectable hydrogel combination therapies for myocardial infarction are extensively investigated in preclinical studies. The surveyed literature includes investigations on cellular therapies, cytokine therapies, pharmacotherapies, extracellular vesicular therapies, and nucleic acid therapies. Additionally, there is an exploration of the combined use of these therapies in a multitherapy approach.

Although there have been numerous preclinical studies, clinical investigations on hydrogel-based treatments for myocardial infarction remain scarce [10, 11]. However, there has been a particular focus on hydrogel combined with stem cell therapies. Building upon previous systematic review and meta-analysis literature, our study delves deeper into hydrogel-based therapeutic approaches [12]. We aimed to analyze the effects of combining hydrogel with various therapies on cardiac function and morphology following myocardial infarction. This analysis provides valuable insights for future research and supports the clinical application of hydrogel combination therapy.

## Materials and methods

### Protocols and registration

This meta-analysis adhered to the Preferred Reporting Items for Systematic Evaluation and Meta-Analysis (PRISMA) guidelines (Supplementary Table I). The review protocol was registered on PROSPERO (CRD42023401702).

### Search strategy and data sources

For this meta-analysis, relevant research literature was sourced from PubMed (National Library of Medicine, 2021/03/01), Web of Science (via Clarivate Analytics), Scopus (via Elsevier 1788–2021/03/01), and Cochrane Central Register of Controlled Trials (via The Cochrane Library, 2021/03/01). The search strategy for PubMed is presented in Supplementary Table II.

### Study eligibility

Two independent evaluators (H.G. and T.Y.) initially assessed the titles and abstracts of the literature against the inclusion and exclusion criteria (Supplementary Table III). Afterward, both evaluators conducted a comprehensive full-text review. This review focused on the outcomes of incorporating injectable hydrogels with various therapies (cellular therapy, pharmacotherapy, cytokine therapy, extracellular vesicular therapy, nucleic acid therapy, and polypharmacy) in animal models of myocardial infarction induced by LAD ligation, with the goal of evaluating improvements in cardiac function and morphology following treatment. To ensure the consistency of study protocols, we required a minimum follow-up duration exceeding 1 week in the included studies [12, 13]. The infarct model was precisely defined as an animal model established using left anterior descending branch ligation, providing reliable and consistent results. Studies reporting immunogenic reactions or solely involving hydrogel injection without other therapies were excluded. There were no language or publication date restrictions in the literature inclusion criteria.

The primary outcome indicators in this study include left ventricular ejection fraction and fractional shortening. To be included in the literature review, the studies must present at least one of these primary outcome measures. Additionally, the secondary outcome indicators encompass left ventricular end-systolic volume (ESV), end-diastolic volume (EDV), end-systolic diameter (ESD), end-diastolic diameter (EDD), infarct size, and anterior wall thickness, covering both cardiac functional and morphological parameters. In cases where the necessary data were missing in the literature but evidence suggested that the primary outcome measures were collected, we contacted the respective authors via email. They were given a two-week period from the date of the email to provide the required information.

### Data extraction

The relevant data for this analysis were extracted using a standardized approach. This included gathering information on the sample size of the experimental animals and measuring the following parameters: baseline, hydrogel group, and combined protocol group for ejection fraction; baseline, hydrogel alone, and combined protocol group for fractional shortening; hydrogel alone and combined protocol group for left ventricular end-systolic diameter, left ventricular end-diastolic diameter, left ventricular end-systolic volume, left ventricular end-diastolic volume, infarct area, and anterior ventricular wall thickness. When data appeared only in graphical format, manual extraction was performed using Image J software. To ensure data precision, both SS. Q and JL. Y independently conducted the extraction. In cases where discrepancies arose in the extraction outcomes, a separate re-measurement was performed to maintain data accuracy.

The literature data were extracted in the format of mean and standard deviation. In cases where the mean standard deviation was not provided, conversion was performed using standard errors and confidence intervals, following the guidelines of the Cochrane Collaboration Network.

The quality of articles was evaluated using the Heyland Methodological Quality Score (MQS) [14]. This score, which could reach a maximum of 18 points, was distributed among criteria such as randomization, analysis, blinding, selection, group comparability, degree of follow-up, treatment regimen, combined interventions, and outcome reporting, with each criterion receiving 2 points.

The risk of bias was assessed using SYRCLE’s Risk of Bias in Animal Testing tool [15]. The assessed elements included sequence generation, implementation, detection, attrition, and reporting bias. If no data were available, an “unclear” designation was assigned. A “high risk” designation was given when the methodology potentially compromised the accuracy of the results, and a “low risk” designation was assigned when the methodology was deemed not to influence the outcomes.

### Statistical analysis

The analysis focused on changes in baseline values for the hydrogel injection and hydrogel combination treatment groups following myocardial infarction, particularly investigating left ventricular functional and morphological outcomes. The data were presented as mean ± standard deviation (SD). In cases where only mean and standard error were provided, we converted the standard error to standard deviation using the sample size. If a study included multiple intervention or control groups, we combined relevant outcome indicator groups, following established literature methodologies to minimize analysis errors [16]. The pooled analysis was conducted using the inverse variance method and a random effects model in the data software. A 95% confidence interval was adopted, with significance set at *P* < 0.05.

The forest plots presented the relative treatment effects and their 95% confidence intervals (CIs) for each outcome indicator across individual studies, different combination therapy types, and the overall random-effects meta-analysis for each parameter investigated. To account for study heterogeneity, the analyses were stratified based on animal size. The initial data analysis was performed using Review Manager (RevMan) 5.3 (Nordic Cochrane Centre in collaboration with the Cochrane Collaboration in Copenhagen, Denmark).

In the priori subgroup analysis, we examined various variables, including combination therapy (encompassing multitherapy or monotherapy), subtype of hydrogel source, sex of the animals, intervals post-MI for both follow-up and treatment, Methodological Quality Score (MQS), general subtype of the animals, and specifically murine small animal subtype. For continuous variables such as cell dose, duration, and MQS, dichotomous subgroup analyses were conducted using the median value obtained from all studies included in the meta-analysis. Meta-regression analyses, employing STATA MP software v17 (StataCorp in College Station), were carried out when the study count reached or exceeded three, with a significance threshold of *P* < 0.05, to determine the impact of subgroup variations.

The heterogeneity among the included studies was evaluated using the Cochran Q statistic, with statistical significance determined at *P* < 0.10. The interpretation of the I^2^ values was as follows: I^2^ < 50%, indicating moderate heterogeneity; 50% ≤ I^2^ ≤ 75%, indicating substantial heterogeneity; I^2^ > 75%, indicating considerable heterogeneity. Further sensitivity analyses were performed to investigate potential sources of heterogeneity by systematically excluding individual trials and utilizing different effect models (STATA MP v17).

Publication bias was assessed through a combination of visual examination of funnel plot results and statistical tests, including Begg’s and Egger’s tests, with *P* < 0.05 considered as evidence of a small study effect. To meet standard literature requirements, at least 9 studies were included in the assessment of publication bias [17].

## Results

### Search results

**Fig. 1:**
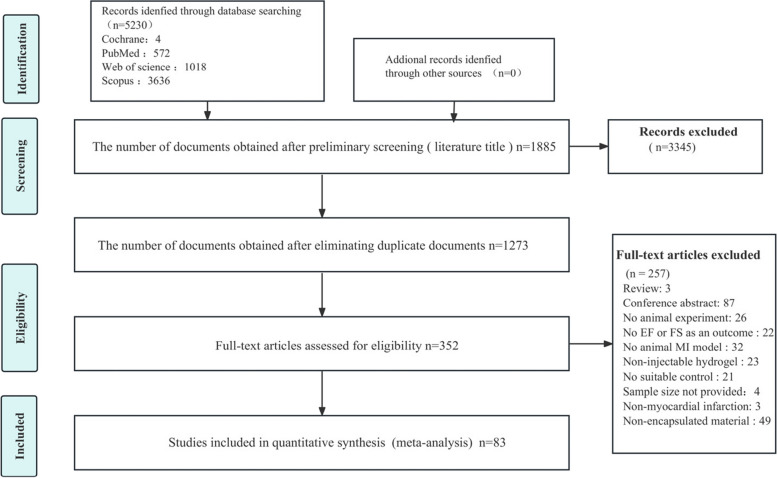
Flowchart of the review process for the meta-analysis

The PRISMA review flowchart is depicted in Fig. 1. Initially, the search of PubMed, Web of Science, Scopus, and Cochrane databases resulted in 5230 relevant articles. After screening the titles, 3345 articles were deemed irrelevant and discarded. Duplicates were eliminated in the remaining 1885 articles that underwent title and abstract review, leaving 352 articles. After evaluating the full text of these 352 articles, 269 were excluded as they did not meet the inclusion and exclusion criteria. As a result, 83 articles were deemed suitable for analysis.

### Study characteristic

Table 1 displays the characteristics of the included studies. The meta-analysis primarily focused on murine small animal models (*N* = 73; 88%), with rats (*N* = 54; 65.1%) and mice (*N* = 19; 22.9%) being the most prevalent. Other animal models consisted of rabbits (*N* = 3; 3.6%), sheep (*N* = 2; 2.4%), and pigs (*N* = 6; 7.2%). Notably, one study utilized both rat and sheep models. Among the selected studies, hydrogels fell into two categories: those of natural origin (*N* = 44; 53%) and chemically synthesized ones (*N* = 39; 47%). Hydrogels derived from natural material backbones were classified as natural origin. Combination therapies were predominantly represented by monotherapy (*N* = 62; 74.7%) and polytherapy (*N* = 21; 25.3%), each further categorized based on variations in therapeutic effects. Monotherapy included cell therapy (*N* = 32; 38.6%), cytokine therapy (*N* = 14; 16.9%), drug therapy (*N* = 10; 12%), extracellular vesicle therapy (*N* = 4; 4.8%), and nucleic acid therapy (N = 2; 2.4%). Most studies utilized male animal models (*N* = 68; 81.9%), while 12 studies (14.5%) incorporated female models. All animal models underwent the left coronary artery ligation method to induce myocardial infarction, ensuring consistent and reliable results. The majority of the animal studies had a 4-week follow-up period after intracardiac injection of the therapeutic hydrogel, followed by autopsy (*N* = 65; 78.3%). In larger animals such as sheep and pigs, the typical follow-up period was extended to 8 weeks, with the longest study having a follow-up period of 52 weeks. In 73 studies (88%), the hydrogel injection occurred immediately after myocardial infarction modeling. The funding sources varied, with 58 studies (69.9%) receiving joint funding from institutions and companies, 19 studies (22.9%) solely funded by institutions, and 6 studies (7.2%) solely funded by companies. One study (1.1%) did not report its funding source. Geographically, the majority of the studies were based in China (46) and the United States (17). Other contributions included Canada (4), Taiwan, China (3), Iran (2), Japan (2), Korea (2), and Singapore (2), with Denmark, France, Germany, and Italy each having contributed one study.

**Table 1:** Characteristics of included studies

	Reference (study, year)	Animal	Total N	Age	Weight(SD)	Follow-up	Timing of Treatment (post-MI)	Baseline EF or FS (post-MI)	Encapsulation materials	Hydrogel	Funding Source	Setting
Rufaihah et al. 2013 [18]	Combined group	Rats	7F	Not reported	250 g	4w	0	Not reported	VEGF-A	PEGylated fibrinogen hydrogel	Agency	Singapore
	Hydrogel group		7F									
Rufaihah et al. 2017 [19]	Combined group	Rats	10 M	Not reported	250-300 g	4w	0	Not reported	VEGF+ANG-1	polyethylene glycol-fibrinogen (PF) hydrogels	Agency/Idustry	China
	Hydrogel group		10 M									
Rocker et al. 2022 [20]	Combined group	Mice	4	Not reported	24–28 g	4w	0	Not reported	VEGF+IL-10 + PDGF	S-GC-PNIPAM hydrogel	Agency	USA
	Hydrogel group		4									
Steele et al. 2017 [21]	Combined group	Rats	10 M	Not reported	250–300 g	4w	0	Not reported	HGFdf	PEG-PNIPAM-P1 hydrogel	Agency	USA
	Hydrogel group		10 M									
Steele et al. 2020 [22]	Combined group	Rats	12 M	Not reported	250-300 g	4w	0	Not reported	HGFdf+ESA 1α	hydhyaluronic acid poly(ethyleneglycol) poly(lactic acid) hydrogel	Agency	USA
	Hydrogel group		12 M									
Steele et al. 2020 [22]	Combined group	Sheep	7 M	Not reported		8w	0	Not reported	HGFdf+ESA 1α	hydhyaluronic acid poly(ethyleneglycol) poly(lactic acid) hydrogel	Agency	USA
	Hydrogel group		7 M									
Chow et al. 2017 [23]	Combined group	Rats	5 M	8 weeks		10w	0	EF = 45.4 ± 7.8	Human Induced Pluripotent Stem Cell-Derived Cardiomyocyte	PEG hydrogel	Agency/Idustry	UK
	Hydrogel group		6 M									
Gaffey et al. 2015 [24]	Combined group	Rats	10 M	Not reported	250-300 g	4w	0	Not reported	Rat bone marrow-derived endothelial progenitor cells	hyaluronic acid hydrogel		USA
	Hydrogel group		10 M									
Hakravarti et al. 2018 [25]	Combined group	Mice	4F	12 weeks		3w	0	Not reported	Human adipose-derived stem cells	gelatin methacrylamide (GelMA)	Agency	USA
	Hydrogel group		4F									
Paul et al. 2014 [26]	Combined group	Rats	7 M	Not reported	200-250 g	2w	0	EF = 36.6	Graphene oxide +VEGF	GelMA hydrogel	Agency/Idustry	USA
	Hydrogel group		7 M									
Qian et al. 2022 [27]	Combined group	Rats	8 M	Not reported	Not reported	4w	0w	Not reported	platelet concentrates including PRP	ALG-HA hydrogels	Agency/Idustry	China
	Hydrogel group		8 M									
Xu et al. 2017 [28]	Combined group	Rats	6 M	Not reported	225-250 g	4w	1w	Not reported	rat bone-marrow mesenchymal stem cells	chitosan hydrogel	Agency	China
	Hydrogel group		6 M									
Follin et al. 2018 [29]	Combined group	Rats	13 M	Not reported	266 ± 15 g	4w	0w	EF = 45.04 ± 9.08	adipose-derived Mesenchymal stem cell	Alginate Hydrogel	Agency	Denmark
	Hydrogel group		10 M									
Fu et al. 2022 [30]	Combined group	Rats	10 M	8 weeks	200–250 g	4w	0w		bFGF	CMCS-S-S-Py and rBSA hydrogel	Agency/Idustry	China
	Hydrogel group		10 M									
Purcell et al. 2018 [31]	Combined group	Pigs	7 M	Not reported	20,000 g	4w	0w	Not reported	rTIMP-3	hyaluronic acid (HA)-based hydrogel	Agency	USA
	Hydrogel group		7 M									
Purcell et al. 2014 [32]	Combined group	Pigs	7 M	Not reported	25,000 g	4w	0w	Not reported	rTIMP-3	Dextran sulphate (DS) / ALD modification of its diol groups hydrogels	Agency/Idustry	USA
	Hydrogel group		7 M									
Cimenci et al. 2022 [33]	Combined group	Mice	7F	Not reported	Not reported	5w	0w	Not reported	fisetin	thermoresponsive collagen hydrogel	Agency/Idustry	Canada
	Hydrogel group		7F									
Fan et al. 2019 [34]	Combined group	Rats	12F	Not reported	200-220 g	4w	0w	Not reported	GST-TIMP-bFGF	collagen-GSH hydrogel	Agency/Idustry	China
	Hydrogel group		12F									
Chen et al. 2018 [35]	Combined group	Rats	11 M	Not reported	350-375 g	5w	0w	Not reported	EPCs EVs	adamantane-modified HA (Ad-HA) and β-cyclodextrin-modified HA (CD-HA) shear-thinning hydrogel (STG)	Agency/Idustry	USA
	Hydrogel group		10 M									
Kim et al. 2020 [36]	Combined group	Mice	9 M	7 weeks	20-22 g	4w	0w	Not reported	MSC	gelatin–hydroxyphenyl propionic acid (GH) hydrogels	Agency	Korea
	Hydrogel group		9 M									
Han et al. 2019 [36]	Combined group	Rats	5 M			4w	0w	Not reported	UMSC exosomes	(PA-GHRPS and NapFF) PGN hydrogel	Agency/Idustry	China
	Hydrogel group		5 M									
Chen et al. 2013 [37]	Combined group	Rats	8 M	6 weeks	200-250 g	4w	0w	EF = 37.9	allogeneic bone marrow mononuclear cells	hyaluronan (HA) hydrogel	Agency/Idustry	Taiwan of China
	Hydrogel group		8 M									
Chen et al. 2014 [38]	Combined group	Pigs	7	Not reported	Not reported	8w	0w	EF = 45.8	bone marrow mononuclear cells	hyaluronan (HA) hydrogel	Agency/Idustry	Taiwan of China
	Hydrogel group		8									
Projahn et al. 2014 [39]	Combined group	Mice	8 M	Not reported	25–26 g	4w	0w	Not reported	CXCL12 (S4V)、Met-CCL5	sP(EO-stat-PO)/oxidation of thiolated (FDH) / PEG-diacrylate(SDH)	Agency/Idustry	Germany
	Hydrogel group		8 M									
Mathieu et al. 2012 [40]	Combined group	Rats	9F	Not reported	180-190 g	8w	0w	Not reported	rat bone-marrow mesenchymal stem cells	Silanized-Hydroxypropyl Methyicellulose hydrogel	Agency/Idustry	France
	Hydrogel group		7F									
Xu et al. 2014 [41]	Combined group	Rats	5F	Not reported	150-200 g	4w	0w	Not reported	Rat bone marrow mesenchymal stem cells	Col-SH/OAC-PEG-OAC hybrid hydrogels	Agency/Idustry	China
	Hydrogel group		5F									
Chen et al. 2017 [42]	Combined group	Mice	6 M	8-11 weeks		2w	0w	Not reported	Curcumin+NO	FFE-ss-ERGD hydrogels	Agency/Idustry	China
	Hydrogel group		6 M									
Awada et al. 2017 [43]	Combined group	Rats	8 M	6-7 weeks	175-225 g	8w	0w	Not reported	TIMP-3 + FGF-2+ SDF-1α	fibrin gel hydrogels	Agency	USA
	Hydrogel group		8 M									
Wang et al. 2010 [44]	Combined group	Rats	8F	6 weeks		4w	0w	Not reported	bFGF	chitosan hydrogel	Agency/Idustry	China
	Hydrogel group		8F									
Wang et al. 2012 [45]	Combined group	Rats	32F	6 weeks		4w	1w	Not reported	mouse embryonic stem cells	OPF hydrogels [fumaryl chloride and poly(ethylene glycol) (PEG)]	Agency	USA
	Hydrogel group		24F									
Wang et al. 2014 [46]	Combined group	Rats	20 M	Not reported	250 ± 10 g	4w	0w	Not reported	CD29 Rat brown adipose-derived stem cells	chitosan hydrogel	Agency/Idustry	China
	Hydrogel group		20 M									
Ding et al. 2020 [47]	Combined group	Rats	6 M	Not reported	250 g	4w	0w	EF = 42.4 FS = 21.9	mesenchymal stem cells	methacrylate hyaluronic acid hydrogel	Agency/Idustry	China
	Hydrogel group		6 M									
Li et al. 2018 [48]	Combined group	Mice	4 M	8–10 weeks	20 ± 5 g	4w	0w	Not reported	Mouse Induced pluripotent stem (MiPS) cells	folic acid (FA) Hydrogel	Agency/Idustry	China
	Hydrogel group		4 M									
Zhu et al. 2017 [49]	Combined group	Rats	7 M	8-10 weeks	200-250 g	4w	0w	Not reported	bFGF	Dex-PCL-HEMA/PNIPAAm hydrogel	Agency/Idustry	China
	Hydrogel group		7 M									
Cohen et al. 2014 [50]	Combined group	Mice	5 M	10 weeks	25-30 g	2w	0w	Not reported	Neuregulin-1β	hyaluronate hydrogel	Agency	USA
	Hydrogel group		5 M									
Cohen et al. 2020 [22]	Combined group	Sheep	6 M	26-30 weeks	35,000-40,000 g	8w	0w	Not reported	Neuregulin-1β	hyaluronate hydrogel	Agency	USA
	Hydrogel group		4 M									
Ding et al. 2020 [51]	Combined group	Rats	5 M	8 weeks	200-250 g	4w	0w	Not reported	catalase + hyperbranched polymers	Reactive Oxygen Species Scavenging and O2 Generating Injectable Hydrogel/hyaluronic acid (HA-MA)	Agency/Idustry	China
	Hydrogel group		5 M									
Zhou et al. 2021 [52]	Combined group	Rats	10 M	Not reported	250 ± 10 g	4w	0w	Not reported	melanin	Alginate (Alg) hydrogels	Agency/Idustry	China
	Hydrogel group		10 M									
Chen et al. 2021 [53]	Combined group	Rats	4 M	6-8 weeks	220-250 g	4w	0w	Not reported	Astragaloside IV	(PEGDA-PBA) - (HA-SH) hydrogel	Agency/Idustry	China
	Hydrogel group		4 M									
Chen et al. 2014 [54]	Combined group	Rabbits	7 M	Not reported	2200-2600 g	4w	1w	EF = 56.13 ± 7.51	Rabbit bone marrow stem cells	α-cylcodextrin/MPEG–PCL–MPEG hydrogel	Idustry	China
	Hydrogel group		7 M									
Wu et al. 2011 [55]	Combined group	Rats	10	Not reported	200-250 g	5w	1w	FS = 28.9	VEGF	PVL-b-PEG-b-PVL/aliphatic polyester hydrogel (HG)	Agency/Idustry	Canada
	Hydrogel group		11									
Khan et al. 2022 [56]	Combined group	Rats	9F	Not reported	250-300 g	4w	0w	Not reported	hAMSC/amniotic stromal mesenchymal stem cells	chitosan and hyaluronic acid (C/HA) based hydrogel	Agency	Canada
	Hydrogel group		10F									
Cheng et al. 2012 [57]	Combined group	Mice	8 M	10-12 weeks	Not reported	3w	0w	EF = 31.8	Cardiosphere-derived cells (CDCs)	hyaluronan and porcine gelatin hydrogel	Agency	USA
	Hydrogel group		8 M									
Zhu et al. 2022 [58]	Combined group	Rats	6 M	Not reported	200 g	4w	0w	Not reported	Mesenchymal stem cell and 68Ga3+ cations	GNR@SNs/PLGA-PEG-PLGA hydrogel	Agency/Idustry	China
	Hydrogel group		6 M									
Wu et al. 2023 [59]	Combined group	Mice	6 M	8 weeks	22-25 g	4w	0w	EF = 37.55 FS = 19	SDF-1/CMs	dECM hydrogel	Agency/Idustry	China
	Hydrogel group		6 M									
Montazeri et al. 2020 [60]	Combined group	Rats	5 M	8 weeks	250–300 g	4w	0w	Not reported	vascular endothelial growth factor (VEGF)/ hESC-CPC-derived cardiomyocytes	fibrin hydrogel	Agency/Idustry	Iran
	Hydrogel group		5 M									
Reis et al. 2015 [61]	Combined group	Rats	7 M	Not reported	200–250 g	6w	0w	Not reported	prosurvival angiopoietin-1–derived peptide, QHREDGS	chitosan-collagen hydrogel	Agency/Idustry	Canada
	Hydrogel group		7 M									
Liu et al. 2020 [62]	Combined group	Mice	8	Not reported	Not reported	4w	0w	Not reported	bone marrow-derived Mesenchymal stem cell	chitosan (CS) thermosensitive hydrogel	Agency/Idustry	China
	Hydrogel group		8									
Vong et al. 2018 [63]	Combined group	Mice	6 M	7-8 weeks	32–35 g	4w	0w	Not reported	NO	PMNT-PEG-PMNT PArg-PEG-PArg+PAAc	Agency/Idustry	Japan
	Hydrogel group		6 M									
Gao et al. 2020 [64]	Combined group	Mice	5 M	8-10 weeks	Not reported	4w	0w	Not reported	Mesenchymal stem cells	bioglass (BG)/γ-polyglutamic acid/chitosan hydrogel	Agency/Idustry	China
	Hydrogel group		5 M									
Ciuffreda et al. 2018 [65]	Combined group	Rats	10F	Not reported	Not reported	4w	1w	EF25.14 ± 4.15 FS30.90 ± 4.68	rat bone-marrow MSC	polyethylene glycol (PEG)-based hydrogel containing heparin (H-HG)	Agency	Italy
	Hydrogel group		10F									
Chang et al. 2016 [66]	Combined group	Pigs	6 M	20 weeks	22.26 ± 0.78 kg	8w	0w	EF = 47.5	human cord blood mononuclear cells	hyaluronan (HA) hydrogel	Agency/Idustry	Taiwan of China
	Hydrogel group		6 M									
Chen et al. 2019 [67]	Combined group	Rats	10 M	Not reported	Not reported	4w	0w	Not reported	IL-10	AdHA and CDHA hydrogel	Agency/Idustry	USA
	Hydrogel group		8 M									
Song et al. 2014 [68]	Combined group	Rats	10 M	6 weeks	Not reported	4w	0w	Not reported	stem cell homing factor (SDF-1) angiogenic peptides	Biomimetic hyaluronic acid based hydrogel	Agency	Korea
	Hydrogel group		10 M									
Qi et al. 2020 [69]	Combined group	Rats	8 M	Not reported	200-250 g	4w	1w	Not reported	Bioglass	Alginate hydrogel	Agency/Idustry	China
	Hydrogel group		8 M									
Bao et al. 2017 [70]	Combined group	Rats	13 M	Not reported	250 ± 20 g	4w	0w	Not reported	adipose tissue-derived stromal cells	PEG-MEL/HA-SH/GO hydrogels	Agency/Idustry	China
	Hydrogel group		13 M									
Firoozi et al. 2020 [71]	Combined group	Rats	6 M	Not reported	280-350 g	4w	0w	Not reported	human bone marrow-derived mesenchymal stem cells	(RADA)4-SDKP hydrogel	Agency/Idustry	Iran
	Hydrogel group		6 M									
Shafei et al. 2022 [72]	Combined group	Rats	6 M	12 weeks	250-280 g	4w	0w	FS = 30	microRNA-126/146a mimics in exosomes	alginate hydrogel	Agency	USA
	Hydrogel group		6 M									
Lü et al. 2010 [73]	Combined group	Rats	10 M	6 weeks	Not reported	4w	1w	Not reported	nuclear-transferred embryonic stem cells	temperature-responsive chitosan hydrogel	Agency/Idustry	China
	Hydrogel group		10 M									
Zhu et al. 2022 [58]	Combined group	Rats	10 M	6 weeks	Not reported	4w	0w	Not reported	Umbilical cord mesenchymal stem cells	GelMA-O5/rGO hydrogels	Agency/Idustry	China
	Hydrogel group		10 M									
Bao et al. 2023 [74]	Combined group	Rats	5 M	Not reported	200-220 g	4w	1w	Not reported	superparamagnetic iron oxide (SPIO)	chitosan/β-glycerophosphate (CS/GP) hydrogel	Agency/Idustry	China
	Hydrogel group		5 M									
Wang et al. 2009 [75]	Combined group	Rats	12 M	Not reported	200–250 g	4w	0w	Not reported	recombined human erythropoietin	α-cyclodextrin/MPEG–PCL–MPEG hydrogel	Agency	China
	Hydrogel group		12 M									
Wang et al. 2009 [76]	Combined group	Rabbits	8 M	Not reported	2200–2600 g	4w	0w	Not reported	Bone marrow stem cells	α-cyclodextrin/MPEG–PCL–MPEG hydrogel	Agency/Idustry	China
	Hydrogel group		8 M									
Vu et al. 2015 [77]	Combined group	Pigs	6 M	Not reported	65,000-70,000 g	8w	0w	Not reported	PRP, allopurinol, ascorbic acid and ibuprofen	Gelatin hydrogel	Agency	Singapore
	Hydrogel group		6 M									
Kraehenbuehl et al. 2011 [78]	Combined group	Rats	8 M	Not reported	200-250 g	6w	0w	EF = 52.7	hESC-derived ELC + SMLC+Tβ4	PEG-hydrogels	Agency/Idustry	USA
	Hydrogel group		8 M									
Wan et al. 2014 [79]	Combined group	Rats	14 M	Not reported	200-250 g	4w	0w	Not reported	short-hairpin RNA of angiotensin	(Dex-PCL-HEMA/PNIPAAm) hydrogel	Agency/Idustry	China
	Hydrogel group		14 M									
Wang et al. 2018 [80]	Combined group	Rats	17 M	Not reported	250 ± 20 g	6w	0w	Not reported	plasmid DNA-eNOs + ADSCs	TA-PEG/HA-SH hydrogels	Agency/Idustry	China
	Hydrogel group		16 M									
Lu et al. 2009 [81]	Combined group	Rats	12F	Not reported	Not reported	4w	1w	Not reported	Mouse embryonic stem cells	temperature-responsive chitosan hydrogel	Agency/Idustry	China
	Hydrogel group		13F									
Li et al. 2014 [82]	Combined group	Rats	6 M	Not reported	250 ± 20 g	4w	0w	Not reported	Rat brown adipose-derived stem cells	SWCNTs-modified PNIPAAm hydrogel	Agency/Idustry	China
	Hydrogel group		6 M									
Li et al. 2010 [83]	Combined group	Rabbits	11 M	Not reported	2000–2500 g	4w	1w	Not reported	bone marrow-derived mononuclear cells	Dex-PCL-HEMA/PNIPAAm hydrogel	Agency/Idustry	China
	Hydrogel group		8 M									
Hu et al. 2022 [84]	Combined group	Mice	5 M	8-10 weeks		4w	0w	Not reported	ISL1-Mesenchymal stem cell-Exo	angiogenin-1 hydrogel (Ang-1 gel)	Agency/Idustry	China
	Hydrogel group		5 M									
Li et al. 2021 [85]	Combined group	Pigs	6 M	Not reported	45,000-50,000 g	4w	0w	EF = 35	MSN / miR-21-5p	gelatin hydrogels	Agency/Idustry	China
	Hydrogel group		6 M									
Liu et al. 2021 [86]	Combined group	Rats	20 M	6-8 weeks	200 ± 10 g	4w	0w	Not reported	puerarin + rBMesenchymal stem cell	hyaluronic acid (HA-Tyr) hydrogel	Agency/Idustry	China
	Hydrogel group		20 M									
Wu et al. 2021 [87]	Combined group	Mice	15 M	8 weeks	22-25 g	4w	0w	Not reported	VEGF and B/SF microspheres	alginate based composite hydrogel	Agency/Idustry	China
	Hydrogel group		15 M									
Chen et al. 2020 [88]	Combined group	Mice	20 M	8-12 weeks		4w	0w	EF = 36.4	Rat adipose-derived mesenchymal stem cells	Col-Transglutaminase cross-linked gelatin	Agency/Idustry	China
	Hydrogel group		20 M									
Zhang et al. 2021 [89]	Combined group	Mice	5 M		22-28 g	4w	0w	Not reported	Dendritic cell-derived exosomes (DEXs)	Alginate hydrogel	Agency/Idustry	China
	Hydrogel group		5 M									
Chen et al. 2020 [90]	Combined group	Mice	13 M	6 weeks		4w	0w	Not reported	colchicine	(PLGA–PEG–PLGA) hydrogel	Agency/Idustry	China
	Hydrogel group		11 M									
Xia et al. 2015 [91]	Combined group	Mice	8F	Not reported	Not reported	4w	0w	Not reported	Mouse bone marrow mesenchymal stem cells	poly(NIPAAm-co-HEMA-co-HEMAPCL)-type I collagen hydrogel	Agency	China
	Hydrogel group		8F									
	Lyu et al. 2020											
	Combined group	Rats	5 M	Not reported	220 ± 20 g	4w	0w	Not reported	MSC aggregates (FMAs) hMesenchymal stem cell+PLGA	OHA@HHA hydrogel	Agency/Idustry	China
	Hydrogel group		5 M									
Sakakibara et al. 2002 [92]	Combined group	Rats	10 M	Not reported	250-290 g	4w	1w	FS = 19.8 ± 4.1	bFGF+ fetal cardiomyocyte	gelatin hydrogels	Agency	Japan
	Hydrogel group											
Zheng et al. 2022 [93]	Combined group	Rats	3 M	Not reported	250 ± 20 g	4w	0w	Not reported	bone mesenchymal stem cells (BMesenchymal stem cell)/KLT (a VEGF mimetic peptide with pro-angiogenic effects)	MaHA/B-G-SH/Fe3+ hydrogels	Agency/Idustry	China
	Hydrogel group		3 M									
Zheng et al. 2022 [94]	Combined group	Rats	3 M	Not reported	250 ± 20 g	4w	0w	Not reported	S1P SS-31(plasma enzyme-degradable peptide)/Lipo	PAMB-G-TK/4-Arm-PEG-SG Hydrogels	Agency/Idustry	China
	Hydrogel group		3 M									
Liu et al. 2012 [95]	Combined group	Rats	22 M	Not reported	Not reported	4w	0w	Not reported	Rat adipose-derived mesenchymal stem cells	chitosan hydrogel	Agency/Idustry	China
	Hydrogel group											
Yuan et al. 2019 [96]	Combined group	Rats	8 M	9–10 weeks	200 ± 20 g	4w	0w	Not reported	Mydgf	citrate-containing polyester hydrogel (PPC-ET/PEG Hydrogels)	Agency/Idustry	China
	Hydrogel group											

### Quality and risk of Bias assessment

In assessing the quality of the literature included, a score of ≥11 was considered as indicative of high quality, as determined by the MQS analysis (Supplement Table 4). Out of the literature evaluated, 66 articles (69.5%) met the criteria for high quality. Additionally, only 25 articles (25.8%) explicitly stated the adoption of a blinded analysis when assessing outcome indicators.

The analysis of the risk of bias plot (Supplement Figure 1) revealed a high risk of bias among the literature included. Only 30 trials (36.1%) maintained blinding throughout the outcome measurement process. Most trials did not provide details of a blinding protocol or implement blinding in relation to the animal housing environment and group allocation, indicating a significant risk of bias. None of the trials were excluded from the primary analysis due to concerns regarding quality or bias.

### Effect of injectable hydrogel combination therapy on cardiac function

#### Effects in small animal models

**Fig. 2:**
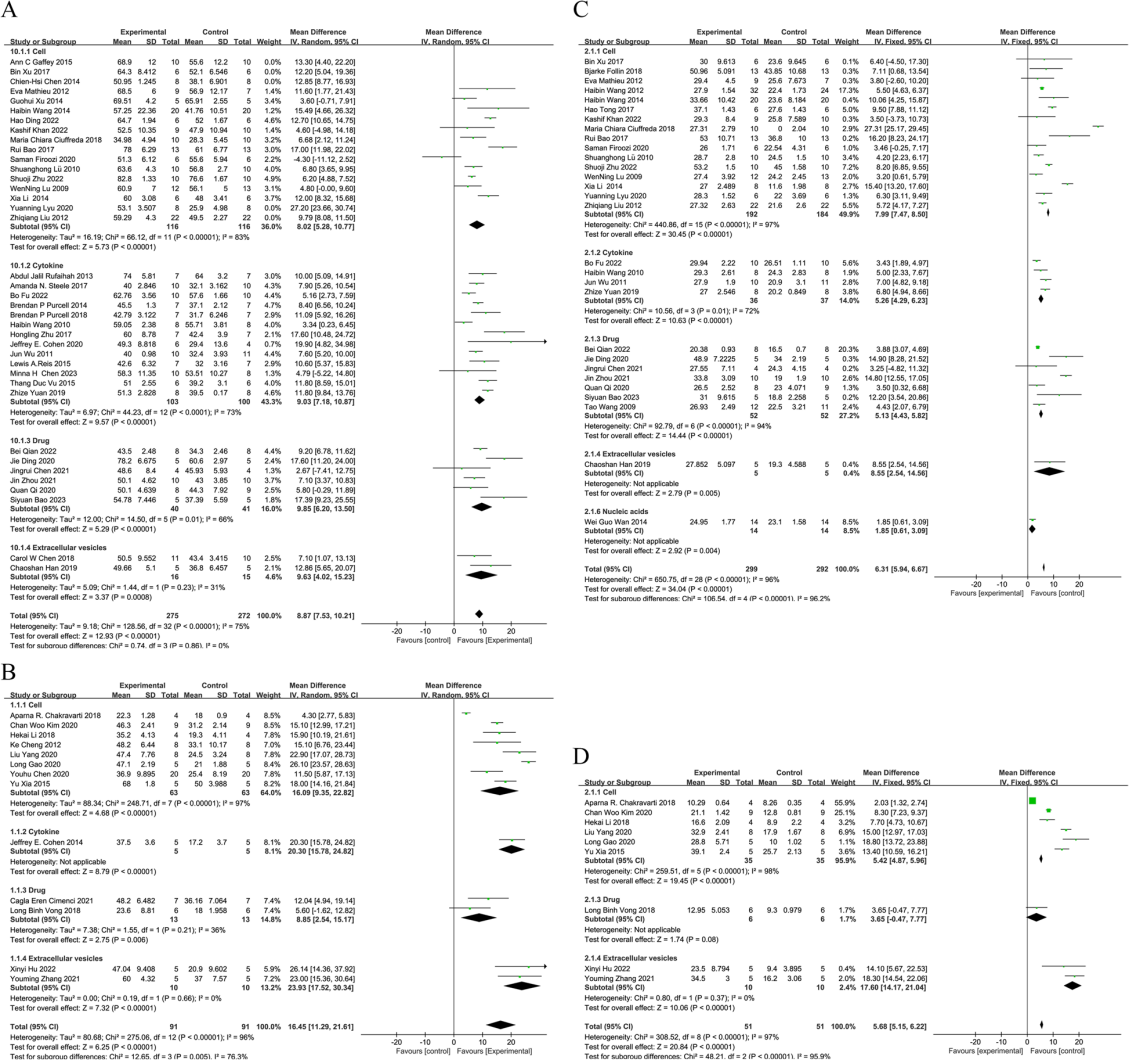
Forest plots of all trials investigating the effect of injectable hydrogel combination therapy on ejection fraction and fractional shortening outcomes in myocardial infarction treatment outcome studies (**a**. Rats EF, **b**. Mice EF, **c**. Rats FS, **d**. Mice FS). Data are expressed as weighted mean differences with 95% CIs, using generic inverse-variance random-effects models. Between-studies heterogeneity was tested by using the Cochran Q statistic (chi-square) at a significance level of *P* < 0.05. Reference numbers for each study can be found in Table 1 and list of references

The use of injectable hydrogel combination therapy resulted in significant improvements in EF (Fig. 2a, b). For rats, the mean difference (MD) was 8.87% [95% confidence interval (CI): 7.53, 10.21], and for mice, the MD was 16.45% [95% CI: 11.29, 21.61]. Similarly, FS (Fig. 2c, d) also showed improvement with the use of injectable hydrogel combination therapy. For rats, the MD was 6.31% [95% CI: 5.94, 6.67], and for mice, the MD was 5.68% [95% CI: 5.15, 6.22]. These improvements were significantly greater than those observed with hydrogels alone. Among the various therapies, cell therapy had the most trials and demonstrated significant enhancements in both EF and FS. For rats, the MD was 8.02% [95% CI: 5.28, 10.77] for EF and 7.99% [95% CI: 7.47, 8.50] for FS. For mice, the MD was 16.09% [95% CI: 9.35, 22.82] for EF and 5.42% [95% CI: 4.87, 5.96] for FS. Extracellular vesicle therapy also showed significant improvements in EF and FS. For rats, the MD was 9.63% [95% CI: 4.02, 15.23] for EF and 8.55% [95% CI: 2.54, 14.56] for FS. For mice, the MD was 23.93% [95% CI: 17.52, 30.84] for EF and 5.68% [95% CI: 5.15, 6.22] for FS. Similar improvements in cardiac function were observed for cytokine therapy and drug therapy. For EF, the MD for rats was 9.03% [95% CI: 7.18, 10.87], and for mice was 20.30% [95% CI: 15.78, 24.82]. For FS, the MD for rats was 5.26% [95% CI: 4.29, 6.23], and for mice was 5.13% [95% CI: 4.43, 5.82]. Only a single study using nucleic acids therapy measured FS as an endpoint. Substantial heterogeneity was observed between studies for both EF (rats: I^2^ = 75%, *p* < 0.0001; mice: I^2^ = 96%, *p* < 0.0001) and FS (rats: I^2^ = 96%, *p* < 0.0001; mice: I^2^ = 97%, *p* < 0.0001). Systematic removal of individual studies did not significantly alter the heterogeneity for either EF or FS. (Supplementary Figure 4a, b).

Regarding the secondary outcomes, the analysis showed significant improvements in ESV for rats (MD = − 0.03 mL [95% CI: − 0.05, − 0.02]) and mice (MD = − 0.09 mL [95% CI: − 0.21, 0.03]). EDV also improved for rats (MD = − 0.03 mL [95% CI: − 0.04, − 0.02]). ESD exhibited improvements for rats (MD = − 0.84 mm [95% CI: − 1.16, − 0.53]) and mice (MD = − 1.23 mm [95% CI: − 2.14, − 0.32]). Similarly, EDD demonstrated improvements for rats (MD = − 0.66 mm [95% CI: − 0.82, − 0.51]) and mice (MD = − 1.13 mm [95% CI: − 3.04, 0.79]). The infarct size also showed positive outcomes with hydrogel combination therapy for rats (MD = − 9.90% [95% CI: − 11.84, − 7.95]) and mice (MD = − 7.64% [95% CI: − 13.67, − 1.62]). Furthermore, wall thickness increased for rats (MD = 0.27 mm [95% CI: 0.12, 0.42]) and mice (MD = 0.07 mm [95% CI: 0.01, 0.12]). These consistent findings indicate the superior treatment outcomes of hydrogel combination therapy compared to sole hydrogel injection (Supplementary Figure 2). Sensitivity analysis of secondary outcome measures also produced relatively robust results. (Supplementary Figure 4c-h).

In addition, multitherapy yielded significant improvements in EF for rats (MD = 12.53% [95% CI: 7.85, 17.21]) and mice (MD = 10.59% [95% CI: 4.32, 16.86]). FS also showed notable improvements for rats (MD = 7.87% [95% CI: 7.00, 8.74]) and mice (MD = 5.88% [95% CI: 4.90, 6.86]). ESD demonstrated reductions for rats (MD = − 1.47 mm [95% CI: − 2.14, − 0.80]) and mice (MD = − 0.18 mm [95% CI: − 0.66, − 0.30]). Similarly, EDD exhibited reductions for rats (MD = − 1.26 mm [95% CI: − 2.51, 0.00]) and mice (MD = − 0.26 mm [95% CI: − 0.46, − 0.07]). Although EDV showed minimal change for rats (MD = − 0.07 mL [95% CI: − 0.18, 0.03]), ESV demonstrated a slight decrease (MD = − 0.07 mL [95% CI: − 0.11, − 0.03]). Infarct size also decreased significantly for rats (MD = − 13.59% [95% CI: − 19.82, − 7.36]) and mice (MD = − 13.44% [95% CI: − 21.66, − 5.22]). Lastly, wall thickness increased for rats (MD = 0.63 mm [95% CI: 0.38, 0.87]) (Supplementary Figure 3).

#### Effects in non- small animal models

**Fig. 3:**
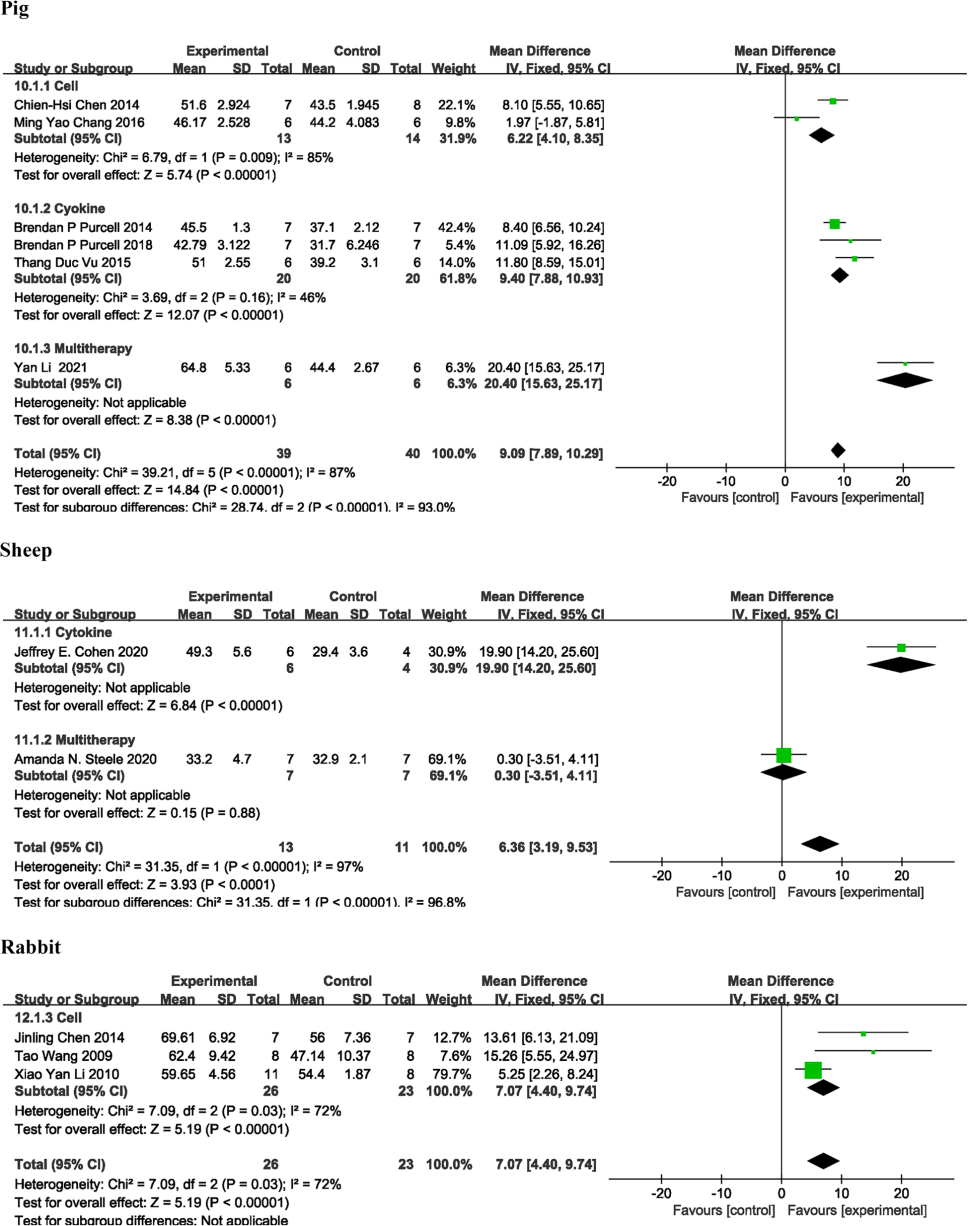
Forest plot to study the effect of injectable hydrogel combination therapy on EF outcomes in a non-murine animal model in the myocardial infarction treatment outcome study. Data are expressed as weighted mean differences with 95% CIs, using generic inverse-variance random-effects models. Between-studies heterogeneity was tested by using the Cochran Q statistic (chi-square) at a significance level of P < 0.05. Reference numbers for each study can be found in Table 1 and list of references

In non-murine studies, the classification and analysis of animal types showed a significant improvement in EF, with an MD of 8.49% [95% CI: 7.46, 9.53]. Among the animal models, the pig model, which had a large sample size, demonstrated the most substantial effect, with an MD of 9.09% [95% CI: 7.89, 10.29]. The sheep (MD = 6.36% [95% CI: 3.19, 9.53]) and rabbit (MD = 7.07% [95% CI: 4.40, 9.74]) models also exhibited significant improvements (Fig. 3). However, secondary outcomes such as FS, ESV, EDV, ESD, EDD, infarct area, and ventricular wall thickness were either not reported or poorly represented, preventing correlation analysis (Tab. 1).

### Subgroup analysis

**Fig. 4:**
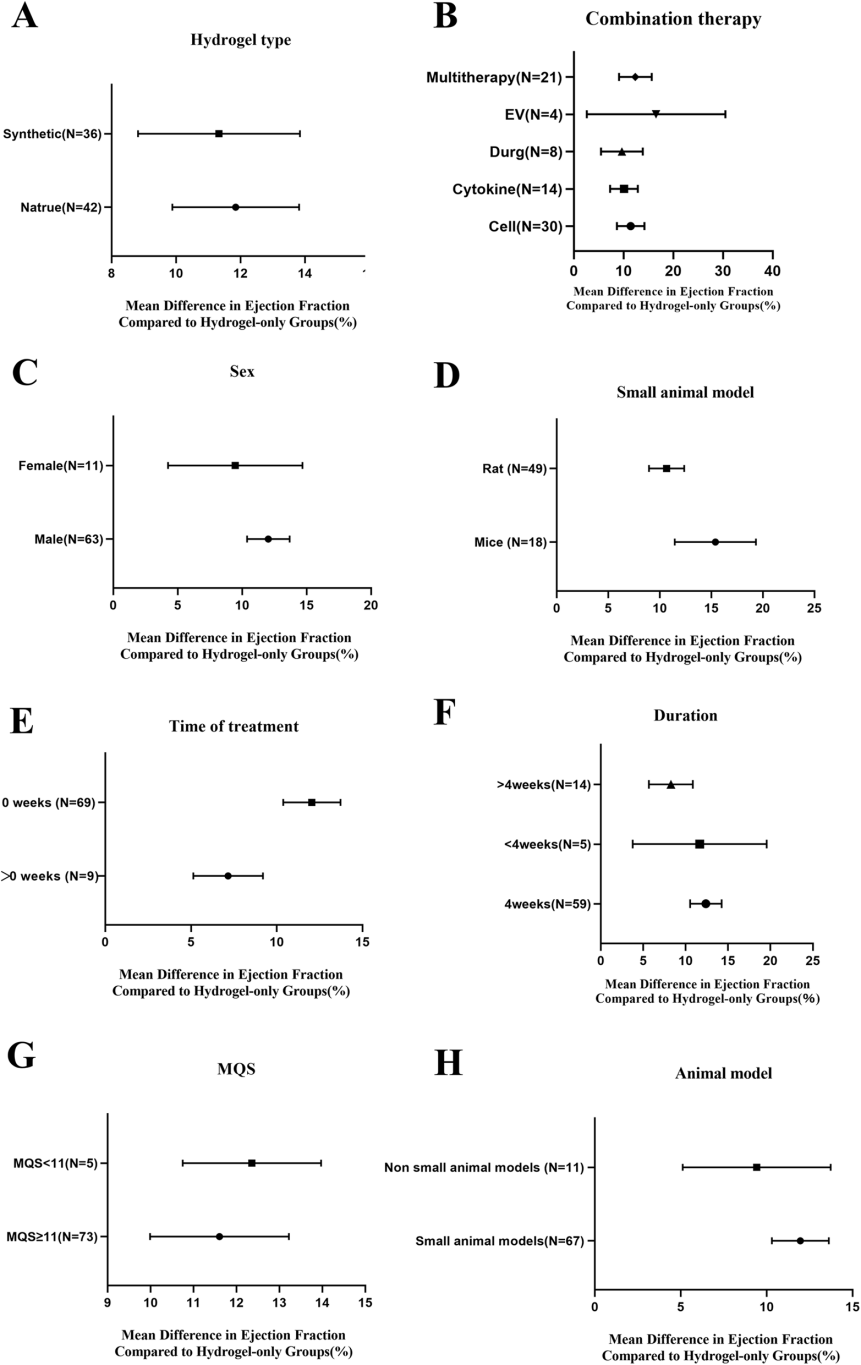
A meta-regression analysis of variables of interest affecting changes in left ventricular ejection fraction. A dichotomous a priori subgroup analysis was performed in a trial examining the effect of hydrogel combination therapy on ejection fraction. Point estimates at each subgroup level are pooled effect estimates for ejection fraction in the hydrogel combination therapy group compared with the hydrogel-only therapy group. **a**. Hydrogel type, **b**. Combination therapy, **c**. Sex, **d**. Small animal model, **e**. Time of treatment, **f**. Durations, **g**. MQS and **h**. Animal model were subjected to subgroup analysis. MQS = Hyland Methodological Quality

This subgroup analysis focused primarily on rat and mouse animal models. Subgroup analysis of combination therapy revealed that extracellular vesicular therapy had the most prominent therapeutic effect, But the larger confidence intervals require more experiments to further validate the actual effect. The second is multitherapy, because it involves many variables, the results are difficult to explain, so it is not included in the main analysis, but it still provides a larger sample size and robust treatment effect. Analyzing follow-up durations highlighted that a 4-week span (*P* < 0.005) yielded the most optimal overall impact, underscoring the significance of follow-up time on outcome indicators, no effect modifications were seen for sex, MQS, animal size, or hydrogels subtype for EF (Fig. 4).

Continuous and subgroup meta-regression analyses demonstrated a significant effect for longer follow-up duration and time of treatment on reducing EF and FS (Fig. 4, Supplement Table 5a-b). For secondary outcomes, continuous meta-regression analyses demonstrated no effect of dose on either ESV, EDV, ESD, EDD, infarct size, or wall thickness. (Supplement Table 5c-h).

In subgroup meta-regression analyses comparing rats and mice, we found that the rat correlation studies (56 articles 65%) had more stable confidence intervals than the mouse correlation studies (17 articles 20%). For secondary outcomes, subgroup meta-regression analyses demonstrated no significant effect of sex, MQS, hydrogel type, Animal model on either ESV, EDV, ESD, EDD, infarct size, or wall thickness (Fig. 4, Supplementary Figure 6–11).

### Publication Bias

**Fig. 5:**
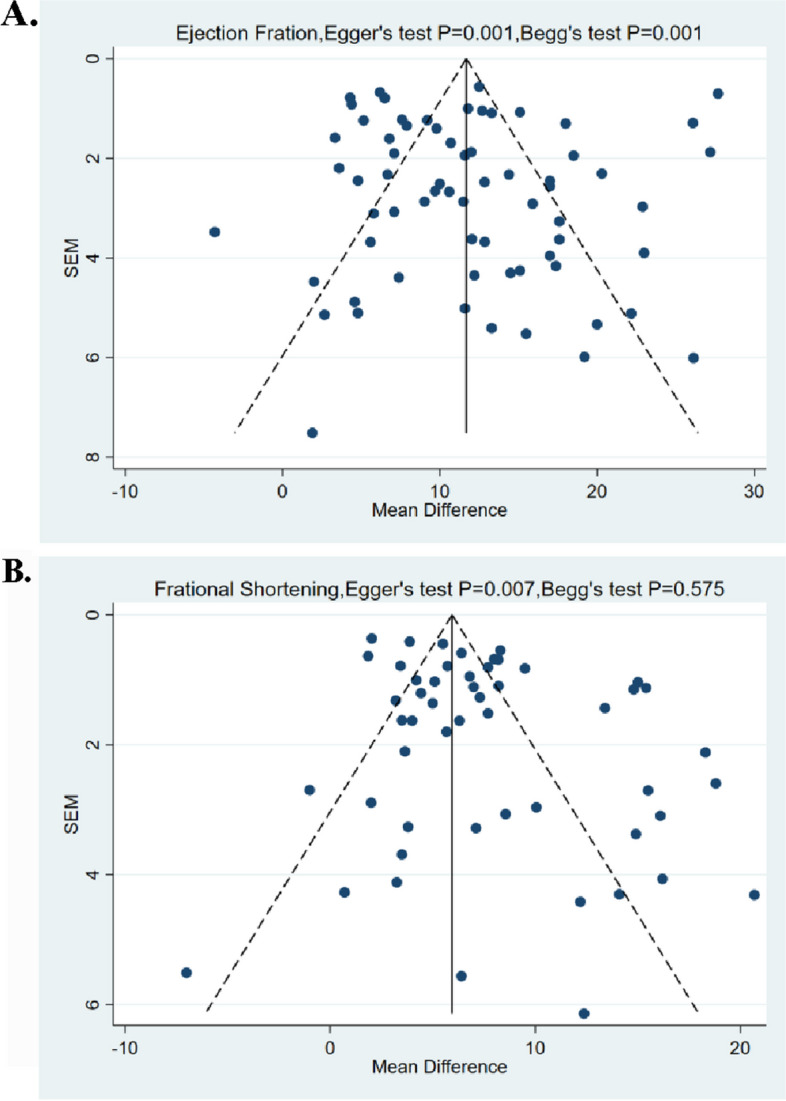
Funnel plots for the effect of Injectable hydrogel-based combination therapy on (A) ejection fraction and (B) fractional shortening in small animal studies

Funnel plot analyses conducted on primary outcomes in a murine small animal model revealed the presence of significant publication bias. The funnel plots depicting EF and FS exhibited an asymmetric distribution. Both Begg’s and Egger’s tests confirmed the presence of publication bias in EF (*P* = 0.001). Additionally, Egger’s test identified bias in FS (*P* = 0.007). Given the discrepancies in the FS results (Begg’s test *P* = 0.575, Egger’s test *P* = 0.007), we rely on Egger’s test due to its slightly higher efficacy in testing (Fig. 5).

Furthermore, the funnel plots for other secondary indicators displayed publication bias in all metrics, except for End Diastolic Volume, which showed no evidence of publication bias (Supplementary Figure 10).

In the case of trials involving large animals, the funnel plot for EF did not portray any noticeable asymmetry (Supplementary Figure 11). Both Egger’s and Begg’s tests yielded non-significant results for publication bias in EF, with reported values of *P* = 0.39 and *P* = 1.000, respectively. Unfortunately, the available data provided insufficient evidence to evaluate publication bias for FS and other secondary metrics in these trials.

## Discussion

Limited systematic evaluations and meta-analyses have been conducted on the therapeutic effectiveness of injectable hydrogels for infarcted myocardium. However, a previous comprehensive review encompassing different biological scaffolds (including injectable hydrogels, microspheres, and patches) combined with stem cell delivery to the infarcted myocardium revealed injectable hydrogels to be superior to other scaffold types [97]. Therefore, our study aimed to further investigate injectable hydrogels. We conducted an analysis of 83 relevant publications, specifically focusing on cardiac morphological and functional measurements that were assessed at the conclusion of the follow-up period in animal models with myocardial infarction induced through left coronary artery ligation. These evaluations encompassed combinations of chemically synthesized hydrogels or naturally derived hydrogels with various therapies, using a control group receiving only hydrogel injections. Our findings demonstrated that the combination of injectable hydrogel and therapy significantly improved primary outcomes, including Ejection Fraction and left ventricular short-axis shortening rate, in comparison to hydrogel injection alone. Additionally, secondary outcomes such as ESD, EDD, ESV, EDV, wall thickness, and infarct size exhibited substantial enhancements. Subgroup analyses indicated a limited body of literature on extracellular vesicle therapy, which poses challenges in drawing definitive conclusions. Cellular therapies, particularly those involving stem cells, consistently demonstrated positive effects. Although the classification of polypharmacy is complex due to the combination of various therapies, it is evident that the combined effect surpasses that of cellular therapy alone. Moreover, the implementation of targeted therapies at each stage of myocardial infarction holds promise as a comprehensive approach, deserving further investigation.

### Monotherapy

#### Cellular therapy

Cell therapy, particularly focusing on stem cell therapy, remains a central area of investigation in combination therapy research [98, 99]. The literature predominantly emphasizes mesenchymal stem cells (MSCs) [62], monocytes [37], embryonic stem cells [45], and human-induced pluripotent stem cells [23]. The integration of stem cell therapy with hydrogel protocols finds applications in the repair of spinal cord injuries [100, 101], osteoarthritis treatment [102], chronic diabetic wound healing [103], cardiovascular disease treatment [104, 105], and hind limb ischemia treatment [106]. MSCs [107] emerge as a promising option due to their ease of isolation, robust proliferative capacity, immunomodulatory ability, and diverse differentiation potential [108]. Many studies encapsulate MSCs from various sources (e.g., bone marrow, adipose tissue, umbilical cord blood) within hydrogels. The enhanced paracrine secretion by MSCs plays a crucial role in the effective repair of cardiac tissue [109]. However, certain research suggests that encapsulation can impact stem cell proliferation and paracrine capability, likely due to limited intercellular interactions within hydrogels, resulting in reduced cytokine secretion [110]. MSCs are often subjected to pre-treatment using physicochemical environments (hypoxia [111], hyperoxia [112], hydrogen sulfide [113]), pharmacological modifications (trimetazidine [114], lipopolysaccharide [115]), and genetic modifications (CXCR4 [116], SDF-1 [117], and HGF [118]) to enhance the paracrine mechanism of MSCs. Yuanning Lyu et al. [119] utilized a combination of human E-cadherin fusion protein (hE-cad-Fc)-encapsulated poly (lactic-co-glycolic acid) (PLGA) particles (hE-cad-PLGA) along with human mesenchymal stem cells (hMSCs) to form 3D cell aggregates, which were then incorporated into hyaluronic acid (HA)-based hydrogels. Incorporating hepatocyte growth factor (HGF)-modified MSCs onto small molecule hydrogels increased Bcl-2 levels, while decreasing Bax and cystein-3 levels, promoting MSC growth and proliferation, and inhibiting apoptosis of cardiomyocytes in the lesioned areas. The pretreatment of MSCs proved more effective than the study without pretreatment. In conclusion, the combination of cell therapy and hydrogel treatment for heart attacks has displayed significant therapeutic effects. This approach offers advantages in promoting tissue regeneration and facilitating healing in areas affected by myocardial infarction through the use of various stem cells or immune cells. To address potential concerns with cell therapy, related studies have explored alternative approaches such as extracellular vesicle therapy or cytokine therapy, which can help mitigate immunogenicity concerns [120].

#### Cytokine therapy

Cytokines (CK) are soluble, low-molecular-weight proteins secreted by various cells and are involved in immune regulation, cell growth, and tissue repair [121]. They encompass different categories, including interleukins, interferons, tumor necrosis factor superfamily, colony-stimulating factors, chemokines, and growth factors. Cytokines play a central role in both the innate and adaptive immune systems, facilitating cell proliferation, activation, and maintaining physiological functions [122]. Jeffrey E. Cohen et al. [22] demonstrated improved ventricular function under ischemic conditions by incorporating epidermal growth factor neuromodulatory protein (NRG) into gelatin hydrogels, which stimulated cardiomyocyte mitogenic activity, reduced apoptosis, and enhanced ischemic ventricular function. Other treatment regimens primarily involve combinations of growth factors such as VEGF, bFGF, and HGF. Considering the complex post-ischemic myocardial environment, cytokine therapy alone may not provide comprehensive repair. Forest plot data indicate that cytokine therapy falls behind other treatments in terms of morphological outcomes following myocardial infarction. As a result, combination therapies or the integration of diverse approaches are often preferred, with further exploration discussed in the subsequent Multitherapy section.

#### Extracellular vesicle therapy

Extracellular vesicles, nanoscale vesicles that result from paracellular secretion, are abundant in the extracellular fluids of animals [123]. Furthermore, it has been demonstrated in related studies that beneficial exosomes can be isolated from plants [124]. These vesicles contain diverse biologically active components and possess properties such as immunomodulation, low antigenicity, and tissue protection [125]. Specifically, exosomes, a subset of these vesicles, carry biologically active biomolecules, including proteins, nucleic acids, lipids, and sugars, granting them a range of biological functions [126]. Their ability to serve as nanocarriers facilitates cell-mediated drug delivery, thereby maximizing therapeutic efficacy. Notably, certain exosomal proteins exhibit selective homing abilities, enhancing the efficiency of delivery [127]. The yield of exosomes is influenced by the type of cells involved, with immune cells often producing consistent and therapeutically potent yields. Clinical trials have successfully utilized exosomes in the diagnosis and treatment of various diseases [128–130].

In the setting of myocardial infarction, it is important to acknowledge that directly injected exosomes may be rapidly cleared due to the myocardial environment. As a result, there has been a growing interest in injectable hydrogel scaffolds to enhance the retention of extracellular vesicles. In a study conducted by Carol W. Chen et al. [35], it was demonstrated that extracellular vesicles, isolated from endothelial progenitor cells and anchored to shear-thinning hydrogels, promote angiogenesis, support functional recovery, and mitigate adverse ventricular remodeling after an infarction. Current research suggests that the therapeutic effects of MSCs are likely due to their paracrine release of cytokines, growth factors, and exosomes, rather than their direct cellular effects [131, 132]. Renae Waters et al. [25] utilized lipid-derived MSCs on methacrylate-based gelatin nanocomposite scaffolds, achieving sustained release of important therapeutic growth factors that stimulate angiogenesis, reduce scarring, and protect the heart. Youming Zhang et al. [89] employed dendritic cell-derived exosomes on alginate hydrogels, revealing enhanced upregulation of Treg cells, polarization of M2 macrophages, reduction of inflammation, and cardiac protection following a myocardial infarction. In summary, extracellular vesicle therapy, which harnesses the paracrine/autocrine mechanisms of MSCs primarily mediated by exosomes, plays a crucial role in mitigating apoptosis, reducing inflammation, promoting angiogenesis, inhibiting fibrosis, and augmenting tissue repair. This meta-analysis highlights the superiority of experiments involving extracellular vesicles compared to other methods in terms of myocardial functional recovery. However, morphological recovery remains limited, and further studies are needed due to the scarcity of literature in this area. Several challenges persist in the development of extracellular vesicles, including the intricate isolation procedures and suboptimal yields [133].

#### Drug therapy

A wide range of medications used in combination with hydrogel for the treatment of myocardial infarction includes natural bioactive drugs such as tanshin and colchicine [90], curcumin [134], compounds (NO [135], Se [136]), and various synthetic products. Bioactive drugs, including curcumin and quercetin, possess strong anti-inflammatory, anti-apoptotic, and tissue repair properties. However, their limited solubility in water hinders efficient delivery through oral or traditional methods. In a study conducted by Cui Yang et al. [136], Se-containing PEG-PPG hydrogels were utilized to reduce pro-inflammatory cytokine secretion, improve myocardial fibrosis, and enhance left ventricular remodeling.

The common characteristic observed among the drugs explored in this section is their demonstrated effectiveness in treating cardiovascular diseases [134, 137]. Nevertheless, their long-term efficacy is often compromised by difficulties in delivery. Hydrogels enable the sustained release of drugs [9], enhancing the local pharmacological benefits while minimizing systemic side effects. This approach is more effective in addressing the prolonged and complex pathological environment [138–140].

#### Nucleic acid therapy

Nucleic acids, such as deoxyribonucleic acid (DNA) and ribonucleic acid (RNA) [141], are vital biomolecules present in living organisms. They are composed of a polymerization of numerous nucleotide monomers. Nucleic acid therapy has been established as a safe and effective approach for treatment. This therapeutic method has shown significant potential in gene regulation, leading to its rapid advancement in cancer treatment as well as the prevention and management of infectious diseases. In particular, mRNA vaccines developed for COVID-19 have played a pivotal role in combating the ongoing viral pandemic [142]. However, despite the promising prospects of nucleic acid therapy, challenges persist in manufacturing, delivery strategies, and targeted site retention.

Nucleic acid therapies, which involve targeting genetic information within the body, hold substantial potential for disease treatment. Unlike conventional therapies with limited effectiveness, nucleic acid approaches have the ability to produce long-lasting effects by modulating genes through suppression, addition, replacement, or editing [97]. However, when applied to cardiovascular diseases, nucleic acid delivery alone is not sufficient due to challenges such as enzymatic degradation, short serum half-life, and low cell transfection efficiency [143]. From a clinical perspective, ensuring effective delivery and retention of nucleic acids at the intended target sites is considered crucial for the success of nucleic acid therapy [9].

Hydrogels serve as promising platforms for nucleic acid therapies, but they require specific chemical modifications to ensure prolonged retention and stability of nucleic acids during treatment, as well as targeted tissue localization and efficient cell delivery. In a rat model, Wei-Guo Wan et al. [79] reported cardioprotective effects by combining a hydrogel with short-hairpin RNA (shRNA). Yan Li et al. [85] developed an injectable hydrogel system for microRNA-21-5p, which showed significant improvements in key indicators and reaffirmed the therapeutic potential of gene/nucleic acid therapy for myocardial infarction.

The microenvironment of the myocardium post-myocardial infarction undergoes a prolonged and complex immune response. Although preclinical studies have provided limited in-depth exploration, drawing definitive conclusions from the small number of existing studies remains challenging [144]. However, these limited findings do suggest the potential of nucleic acid therapy in reducing nucleic acid clearance through hydrogel combinations and effectively restoring damaged myocardial tissue through continuous and substantial gene regulation.

### Multitherapy

Over the past decade, clinical insights and preliminary studies have revealed that a singular approach to treatment falls short of achieving optimal therapeutic outcomes due to the multifaceted nature and physiological intricacies of the disease [145]. As a result, with advancements in drug delivery techniques, the exploration of combination or multitherapy has emerged as a promising avenue of research [145].

Adam J. Rocker et al. [20] adopted a sequential delivery method for three cytokines: vascular endothelial growth factor (VEGF), interleukin-10 (IL-10), and platelet-derived growth factor (PDGF). Initially, VEGF induced angiogenesis and suppressed cardiomyocyte necrosis, followed by the modulation of excessive inflammation by IL-10. The final delivery of PDGF aimed to stabilize the myocardial microenvironment and rejuvenate substantial hemodialysis. This multicytokine approach tailored interventions to the therapeutic demands of various pathological phases. However, while these findings are promising, analysis suggests that the role of PDGF may be limited, indicating the need for further refinement of the regimen. Combining cell therapy with drug therapy has also demonstrated significant therapeutic potential. Enhancing the paracrine impact of MSCs through biomaterial integration can greatly boost therapeutic efficiency, as the full potential of the paracrine function of diverse stem cells is often not realized. Yang Liu et al. [86] incorporated stem cells with puerarin, a natural scavenger of ROS, to mitigate cardiomyocyte damage. Concurrently, in combination with puerarin, bone-derived mesenchymal stem cells increased the secretion of paracrine factors. A similar approach was employed by Shilan Shafei et al. [72], further highlighting the synergistic potential of such combinations.

In summary, strategic combinations of therapies can yield synergistic effects where the combined outcome surpasses the sum of individual contributions [145]. The advantages of combining multiple therapeutic agents outweigh the drawbacks of individual therapies, leading to significant therapeutic benefits [146]. However, it is crucial to ensure effective treatment while also considering biosafety [147]. The future direction of development lies in establishing efficient and safe approaches for combination therapy that undergo repeated research validation and clinical testing.

### Hydrogel source

Injectable hydrogels have been found to be superior to other biological scaffold materials for drug delivery and cardiac implantation [148].

Various experimental results have shown that hydrogel injections can effectively impart specific physical, chemical, and electrical characteristics to the post-infarct myocardial area. This paper categorizes injectable hydrogels into two types: those of natural origin and those that are chemically synthesized. Natural-origin hydrogels, including collagen, fibrin, decellularized materials, chitosan, and alginate, display commendable biochemical properties, bioactivity, and biocompatibility, making them well-suited for in vivo implantation [149]. However, these naturally-derived hydrogels face challenges such as inadequate mechanical properties, consistent degradation rates, antioxidant capacities, and the necessary electrical conductivity for implantation [150]. In a clinical trial involving alginate injectable hydrogels, a higher mortality rate was observed in patients with advanced heart failure who received hydrogel implants compared to those without injections, highlighting significant limitations in the clinical application of natural hydrogels [151]. On the other hand, chemically synthesized hydrogels [152] (such as PNIPAAm-based hydrogels, Aniline-Based Materials, and PEG-based hydrogels) offer superior mechanical properties and stability compared to natural origin hydrogels [153], but often compromise biocompatibility [154]. Subgroup analyses have demonstrated superior functional recovery with natural hydrogels, while chemically synthesized hydrogels excel in morphological recovery. Therefore, the fusion of both categories in the form of hybrid hydrogels emerges as a promising avenue for future research [155].

Hybrid hydrogels provide versatile design options and adaptability to different functions, making them effective in various tissues. Given the distinctive vascular structure, electrical conduction signal function, high metabolism, and high compliance characteristics of myocardial tissue, it is crucial to construct injectable complexes using hybrid hydrogels specifically tailored for myocardial tissue [155]. The findings of this systematic review demonstrate that hybrid hydrogels designed based on the cardiac tissue structure can optimize M2 macrophage polarization, promote angiogenesis, enhance repair response (as indicated by the cardiomyocyte survival rate), thereby reducing infarct size, improving wall thickness, and enhancing cardiac contractility.

### Publication Bias and quality assessment

Consistent with previous research, this analysis identified significant publication bias for the primary outcomes of Ejection Fraction and Fractional Shortening. The bias persisted even after conducting a sensitivity analysis. It is crucial to address this publication bias in order to facilitate genuine clinical trials utilizing injectable hydrogels for myocardial infarction treatment. Evaluation of the SYRCLE risk of bias tool revealed pronounced selection and implementation biases in many studies. Further refinement of research methodologies for myocardial infarction animal models, particularly in interdisciplinary settings, is necessary. To ensure reliable and replicable experimental results, it is imperative to employ blinded protocols for establishing animal models, treatment allocation, and outcome measurement.

Within the reviewed literature, the MQS analysis identified 66 (69.5%) high-quality articles. However, a significant portion of these studies either omitted details in the randomization protocol or did not utilize blinding methods for their experiments. During data collection, studies lacking primary outcome indicators were excluded, resulting in the omission of relevant experimental studies. Future research should prioritize the reporting of echocardiographic parameters and morphological assessments. Comprehensive reporting will not only ascertain the efficacy of experimental protocols but also provide dependable results for subsequent literature reviews and inform future research endeavors. Similar to the challenges observed with nucleic acid therapies discussed earlier, the lack of data compromised the depth of the literature analysis.

### Strengths and limitations

The meta-analysis included 83 papers and provided valuable insights into current research trends. However, there are certain limitations that need to be acknowledged. Firstly, the study primarily focused on murine small animal models due to modeling challenges, and there was limited exploration of large animal models. Therefore, conducting further large animal experiments is necessary to validate the findings. Secondly, it is important to standardize the experimental data in order to facilitate future analyses. Thirdly, the current study faces heterogeneity due to variations in the targeted drug delivery method applied to the heart and the limited number of animal studies available at this stage. This heterogeneity poses a significant barrier to further clinical translation. To address this, standardized large-scale animal experiments are required for validation. Lastly, publication bias was identified in the main outcome indicators, which merits attention.

### Clinical transformation status

With the rapid advancement of hydrogel technology, the clinical use of hydrogel-based combination therapy for various diseases is increasing. While preclinical studies have extensively investigated hydrogel combination therapy for targeted drug delivery and tissue defect repair, there are significant challenges in translating these findings into clinical practice. Hydrogel wound dressings have gained popularity in clinical settings due to their ease of implementation [156, 157]. However, when it comes to diseases that require interventional therapy, conducting effective clinical trials presents substantial difficulties. Therefore, addressing the safety concerns associated with delivery methods is a prerequisite for the progress of injectable hydrogel combination therapy [158].

Clinical trials involving hydrogels in the context of cardiac applications remain limited. The unique structural characteristics of the human heart contribute to the relatively slow progress in developing clinical trials and exploring indications and contraindications. In a randomized controlled trial conducted in 2020, the injection of collagen hydrogel encapsulating mesenchymal stem cells via coronary artery bypass grafting was evaluated [159]. The trial results showed no adverse reactions. Evaluation of the left ventricular ejection fraction at three follow-up time points (3, 6, and 12 months) indicated percentages of 9.14, 9.84, and 9.35% in the hydrogel combined with stem cell treatment group, while the control group exhibited percentages of 4.17, 4.40, and 3.62%. Analysis of cardiac morphological indicators demonstrated no significant changes in myocardial scar tissue in the hydrogel combined with the stem cell group after the 12-month follow-up period. In contrast, both the stem cell treatment group and the control group showed a significant increase in scar tissue. These clinical trial results suggest that the hydrogel combined with stem cell treatment exhibits long-term therapeutic effects, improving cardiac function and morphology.

In conclusion, achieving comprehensive clinical transformation in hydrogel-based combination therapy for myocardial infarction depends on further optimizing the therapeutic approach and enhancing the safety and efficiency of the delivery method.

## Conclusion

This article focuses on evaluating the therapeutic efficacy of injectable hydrogels compared to other types of bio-delivery scaffolds, as determined through a systematic review and meta-analysis. Additionally, this study examines the therapeutic effectiveness of combining injectable hydrogels with different therapies in animal models of myocardial infarction. The findings demonstrate that the combination of injectable hydrogels with other therapies significantly enhances therapeutic outcomes in the ischemic myocardial region, which is crucial for restoring myocardial function and preserving cardiac morphology. The analysis reveals that various combination therapy regimens effectively restore myocardial function and maintain cardiac morphology. Specifically, cellular therapy consistently proves to be therapeutically effective. Moreover, through careful design of functional adaptation and action staging, the utilization of a Multitherapy approach exhibits a synergistic effect, resulting in better outcomes compared to individual therapies alone.

Analyses have demonstrated the close interrelation between the recovery of myocardial function and morphology. However, given the complexity of the recovery process following myocardial ischemia, individual therapies often fall short in achieving efficient restoration of both functional and morphological aspects. Sole reliance on drugs or cellular therapies is inadequate to fully recover damaged myocardium. Therefore, future research should focus on exploring the potential of combined therapies. Furthermore, as the study of combination therapies progresses, it becomes increasingly important to systematically evaluate and conduct meta-analyses of protocols involving injectable hydrogels, which present challenges in subdivision.

In conclusion, hydrogel-based combination therapy demonstrates significant therapeutic effects for myocardial infarction. Based on our analysis of multiple literature sources, we strongly recommend comprehensive monitoring of the therapeutic process and outcome measures in small animal models. Subsequently, large-scale animal experiments should be conducted to validate these effects. Such an approach will provide reliable references for clinical translation and enhance our understanding of hydrogel-based combination therapy. Through a meta-analysis of a wide range of preclinical studies, combined with the findings from conducted clinical trials, it has been demonstrated that hydrogel-based combination therapy yields positive outcomes for the treatment of myocardial infarction.

### Supplementary Information

**Additional file 1: Supplement Table 1.** PRISMA Checklist. PRISMA Checklist* From: *Moher D, Liberati A, Tetzlaff J, Altman DG, The PRISMA Group (2009). Preferred Reporting Items for Systematic Reviews and Meta-Analyses: The PRISMA Statement. PLoS Med 6(6): e1000097. doi:10.1371/journal.pmed1000097. **Supplement Table 2.** Detailed search strategy. **Supplement Table 3.** Study inclusion and exclusion criteria. **Supplement Table 4.** Study quality assessment using the Heyland methodological quality. score. **Supplement Table 5. **A. Ejection Fraction. B. Fractional Shortening. C. End Systolic Diameter. D. End Diastolic Diameter. E. End Diastolic Volume. F. End Systolic Volume. G. Infact size. H. Wall thickness. Continuous a priori subgroup analyses on (A) Ejection Fraction and (B) Fractional Shortening, (C) End Systolic Diameter, (D) End Diastolic Diameter, (E) End Systolic Volume, (F) End Diastolic Volume, (G) Infarct Size, and (H) Wall Thickness in the included studies. β is the slope derived from meta-regression analyses and represents the treatment effect of stem cell embedded scaffolds compared to independent injections of cells for primary and secondary outcomes in the included studies. The residual I^2^ value indicates heterogeneity unexplained by the subgroup and is reported as a percent value, where I^2^ ≤ 50% indicated “moderate” heterogeneity, I²≥ 50% indicated “substantial” heterogeneity, and ≥ 75% indicated “considerable” heterogeneity. P-value significance for heterogeneity was set as P < 0.10. **Supplement Figure 1.** Cochrane risk of bias tool to asses Selection Bias, Performance Bias, Detection Bias, Attrition Bias, and Reporting Bias in studies investigating the effects of stem cell-embedded scaffolds on cardiac repair. Authors’ judgments concerning each risk of bias item are presented as percentages across all included studies. **Supplement Figure 2.** A. End Systolic Diameter. B. End Diastolic Diameter. C. End Systolic Volume. D. End Diastolic Volume. E. Infarct Size. F. Wall Thickness. Forest plots of all trials investigating the effect of hydrogel combination therapy on left ventricular (A) End Systolic Diameter, (B) End Diastolic Diameter, (C) End Systolic Volume, (D) End Diastolic Volume, (E) Infarct Size, and (F) Wall Thickness in the included studies. Pooled effect estimates (diamonds) are shown: one each for trials using hydrogels, patches, microspheres/beads, and their combination (total). Data are expressed as weighted mean differences with 95% CIs, using generic inverse-variance random-effects models. Between-studies heterogeneity was tested by using the Cochran Q statistic (chi-square) at a significance level of P < 0.05. Reference numbers for each study can be found in Table 1 and list of references. **Supplement Figure 3. **A. EF. B. FS. C. End Systolic Diameter. D. End Diastolic Diameter. E. End Systolic Volume. F. Infarct Size. G. Wall Thickness. Forest plots of all trials investigating the effect of hydrogel combination multitherapy on left ventricular (A) EF, (B) FS, (C) End Systolic Diameter, (D) End Diastolic Diameter, (E) End Systolic Volume, (F) Infarct Size, and (G) Wall Thickness in the included studies. Pooled effect estimates (diamonds) are shown: one each for trials using hydrogels, patches, microspheres/beads, and their combination (total). Data are expressed as weighted mean differences with 95% CIs, using generic inverse-variance random-effects models. Between-studies heterogeneity was tested by using the Cochran Q statistic (chi-square) at a significance level of P < 0.05. Reference numbers for each study can be found in Table 1 and list of references. **Supplement Figure 4. **A.EF**. **B.FS. C. End Systolic Diameter. D. End Diastolic Diameter. E. End Systolic Volume. F. Infarct Size. G. Wall Thickness. Sensitivity analysis. A.EF,B.FS,C. End Systolic Diameter, D. End Diastolic Diameter, E. End Systolic Volume, F. Infarct Size and G. Wall Thickness. **Supplement Figure 5. **Meta-regression analysis of variables that may impact changes in Fractional Shortening. Dichotomous a priori subgroup analysis was performed in a trial investigating the effect of injectable hydrogel combination therapy on infarct size. Mean differences in end systolic diameter in the combination regimen treatment group compared to the injectable hydrogel-only treatment group were grouped by A hydrogel type, B combination therapy, C sex, D small animal model, E time of treatment, F Duration, G MQS. **Supplement Figure 6. **Meta-regression analysis of variables that may impact changes in LV End Systolic Diameter. Dichotomous a priori subgroup analysis was performed in a trial investigating the effect of injectable hydrogel combination therapy on infarct size. Mean differences in end systolic diameter in the combination regimen treatment group compared to the injectable hydrogel-only treatment group were grouped by A hydrogel type, B combination therapy, C sex, D small animal model, E time of treatment. **Supplement Figure 7. **Meta-regression analysis of variables that may impact changes in LV End Diastolic Diameter. Dichotomous a priori subgroup analysis was performed in a trial investigating the effect of injectable hydrogel combination therapy on infarct size. Mean differences in end diastolic diameter in the combination regimen treatment group compared to the injectable hydrogel-only treatment group were grouped by A hydrogel type, B combination therapy, C sex, D small animal model, E time of treatment. **Supplement Figure 8.** Meta-regression analysis of variables that may impact changes in LV End Systolic Volume. Dichotomous a priori subgroup analysis was performed in a trial investigating the effect of injectable hydrogel combination therapy on infarct size. Mean differences in end systolic volume in the combination regimen treatment group compared to the injectable hydrogel-only treatment group were grouped by A hydrogel type, B combination therapy, C sex, D small animal model, E time of treatment, F duration. **Supplement Figure 9.** Meta-regression analysis of variables that may impact changes in LV End Diastolic Volume. Dichotomous a priori subgroup analysis was performed in a trial investigating the effect of injectable hydrogel combination therapy on infarct size. Mean differences in end diastolic volume in the combination regimen treatment group compared to the injectable hydrogel-only treatment group were grouped by A hydrogel type, B combination therapy, C sex, D time of treatment, E duration. **Supplement Figure 10.** Meta-regression analysis of variables that may impact changes in Infarct Size. Dichotomous a priori subgroup analysis was performed in a trial investigating the effect of injectable hydrogel combination therapy on infarct size. Mean differences in wall thickness in the combination regimen treatment group compared to the injectable hydrogel-only treatment group were grouped by A hydrogel type, B combination therapy, C sex, D small animal model, E time of treatment, F duration, G MQS, H animal model. **Supplement Figure 11.**Meta-regression analysis of variables that may impact changes in Wall Thickness. Dichotomous a priori subgroup analysis was performed in a trial investigating the effect of injectable hydrogel combination therapy on wall thickness. Mean differences in wall thickness in the combination regimen treatment group compared to the injectable hydrogel-only treatment group were grouped by A hydrogel type, B combination therapy, C sex, D small animal model, E time of treatment, F duration, G animal model. **Supplement Figure 12.** Funnel plot for the effect of Injectable hydrogel combination therapy on (A) End Systolic Diameter, (B) End Diastolic Diameter, (C) End Systolic Volume, (D) End Diastolic Volume, (E) Infarct Size, and (F) Wall Thickness. **Supplement Figure 13.** Funnel plot for the effect of Injectable hydrogel combination therapy on Ejection Fraction in Non-mouse small animal models.

## Data Availability

All data generated or analysed during this study are included in this published article [and its supplementary information files].
