# Supplementary material for: Injectable hydrogel-based combination therapy for myocardial infarction: a systematic review and Meta-analysis of preclinical trials
Source: BMC Cardiovasc Disord. 2024 Feb 21;24:119. doi: 10.1186/s12872-024-03742-0 (PMC10882925; doi:10.1186/s12872-024-03742-0)
Supplement: Supplementary file 1 — Additional file 1: Supplement Table 1. PRISMA Checklist. PRISMA Checklist From: Moher D, Liberati A, Tetzlaff J, Altman DG, The PRISMA Group (2009). Preferred Reporting Items for Systematic Reviews and Meta-Analyses: The PRISMA Statement. PLoS Med 6(6): e1000097. doi:10.1371/journal.pmed1000097. Supplement Table 2. Detailed search strategy. Supplement Table 3. Study inclusion and exclusion criteria. Supplement Table 4. Study quality assessment using the Heyland methodological quality. score. Supplement Table 5. A. Ejection Fraction. B. Fractional Shortening. C. End Systolic Diameter. D. End Diastolic Diameter. E. End Diastolic Volume. F. End Systolic Volume. G. Infact size. H. Wall thickness. Continuous a priori subgroup analyses on (A) Ejection Fraction and (B) Fractional Shortening, (C) End Systolic Diameter, (D) End Diastolic Diameter, (E) End Systolic Volume, (F) End Diastolic Volume, (G) Infarct Size, and (H) Wall Thickness in the included studies. β is the slope derived from meta-regression analyses and represents the treatment effect of stem cell embedded scaffolds compared to independent injections of cells for primary and secondary outcomes in the included studies. The residual I2 value indicates heterogeneity unexplained by the subgroup and is reported as a percent value, where I2 ≤ 50% indicated “moderate” heterogeneity, I²≥ 50% indicated “substantial” heterogeneity, and ≥ 75% indicated “considerable” heterogeneity. P-value significance for heterogeneity was set as P < 0.10. Supplement Figure 1. Cochrane risk of bias tool to asses Selection Bias, Performance Bias, Detection Bias, Attrition Bias, and Reporting Bias in studies investigating the effects of stem cell-embedded scaffolds on cardiac repair. Authors’ judgments concerning each risk of bias item are presented as percentages across all included studies. Supplement Figure 2. A. End Systolic Diameter. B. End Diastolic Diameter. C. End Systolic Volume. D. End Diastolic Volume. E. Infarct Size. F. Wall [file 12872_2024_3742_MOESM1_ESM.docx]

**Supplemental Material**

**Injectable hydrogel-based combination therapy for Myocardial Infarction：**

**a Systematic Review and Meta-Analysis of Preclinical Trials**

Han Gao^1^, Song Liu^1^, Shanshan Qin^1^, Jiali Yang^2^, Tian Yue^2^, Bengui Ye^3^, Yue Tang^4^, Jie Feng^5^, Jun Hou^6^* and Dunzhu Danzeng^1^*

^1^School of Medicine, Tibet Uniersity, Lhasa, Tibet, China.

^2^ School of Life Science and Engineering, Southwest Jiaotong University, Chengdu, Sichuan, China.

^3^ West China School of Pharmacy, Sichuan University, Chengdu, Sichuan, China.

^4^ School of Pharmacy, North Sichuan Medical College,Nanchong,Sichuan,China.

^5^ School of Medicine, Southwest Jiaotong University, Chengdu, Sichuan, China.

^6^ Department of Cardiology, Chengdu Third People's Hospital, Chengdu, Sichuan, China.

**Supplement Table 1. PRISMA Checklist**

| **Section/Topic** | **#** | **Checklist Item** | **Reported on Page #** |
| --- | --- | --- | --- |
| **TITLE** | | | |
| Title | 1 | Identify the report as a systematic review, meta-analysis, or both. | 1 |
| **ABSTRACT** | | | |
| Structured summary | 2 | Provide a structured summary including, as applicable: background; objectives; data sources; study eligibility criteria, participants, and interventions; study appraisal and synthesis methods; results; limitations; conclusions and implications of key findings; systematic review registration number. | 1、2 |
| **INTRODUCTION** | | | |
| Rationale | 3 | Describe the rationale for the review in the context of what is already known. | 2、3 |
| Objectives | 4 | Provide an explicit statement of questions being addressed with reference to participants, interventions, comparisons, outcomes, and study design (PICOS). | 3 |
| **METHODS** | | | |
| Protocol and registration | 5 | Indicate if a review protocol exists, if and where it can be accessed (e.g., Web address), and, if available, provide registration information including registration number. | 3 |
| Eligibility criteria | 6 | Specify study characteristics (e.g., PICOS, length of follow-up) and report characteristics (e.g., years considered, language, publication status) used as criteria for eligibility, giving rationale. | 4 |
| Information sources | 7 | Describe all information sources (e.g., databases with dates of coverage, contact with study authors to identify additional studies) in the search and date last searched. | 4 |
| Search | 8 | Present full electronic search strategy for at least one database, including any limits used, such that it could be repeated. | 4 |
| Study selection | 9 | State the process for selecting studies (i.e., screening, eligibility, included in systematic review, and, if applicable, included in the meta-analysis). | 4 |
| Data collection process | 10 | Describe method of data extraction from reports (e.g., piloted forms, independently, in duplicate) and any processes for obtaining and confirming data from investigators. | 4 |
| Data items | 11 | List and define all variables for which data were sought (e.g., PICOS, funding sources) and any assumptions and simplifications made. | 4 |
| Risk of bias in individual studies | 12 | Describe methods used for assessing risk of bias of individual studies (including specification of whether this was done at the study or outcome level), and how this information is to be used in any data synthesis. | 5 |
| Summary measures | 13 | State the principal summary measures (e.g., risk ratio, difference in means). | 5 |
| Synthesis of results | 14 | Describe the methods of handling data and combining results of studies, if done, including measures of consistency (e.g., I^2^) for each meta-analysis. | 5 |

| **Section/Topic** | **#** | **Checklist Item** | **Reported on Page #** |
| --- | --- | --- | --- |
| Risk of bias across studies | 15 | Specify any assessment of risk of bias that may affect the cumulative evidence (e.g., publication bias, selective reporting within studies). | 5 |
| Additional analyses | 16 | Describe methods of additional analyses (e.g., sensitivity or subgroup analyses, meta-regression), if done, indicating which were pre-specified. | 5 |
| **RESULTS** | | | |
| Study selection | 17 | Give numbers of studies screened, assessed for eligibility, and included in the review, with reasons for exclusions at each stage, ideally with a flow diagram. | 6 |
| Study characteristics | 18 | For each study, present characteristics for which data were extracted (e.g., study size, PICOS, follow-up period) and provide the citations. | 6 |
| Risk of bias within studies | 19 | Present data on risk of bias of each study and, if available, any outcome level assessment (see item 12). | 7 |
| Results of individual studies | 20 | For all outcomes considered (benefits or harms), present, for each study: (a) simple summary data for each intervention group (b) effect estimates and confidence intervals, ideally with a forest plot. | 7 |
| Synthesis of results | 21 | Present the main results of the review. If meta-analyses done, include for each, confidence intervals and measures of consistency. | 8 |
| Risk of bias across studies | 22 | Present results of any assessment of risk of bias across studies (see Item 15). | 8 |
| Additional analysis | 23 | Give results of additional analyses, if done (e.g., sensitivity or subgroup analyses, meta-regression [see Item 16]). | 8 |
| **DISCUSSION** | | | |
| Summary of evidence | 24 | Summarize the main findings including the strength of evidence for each main outcome; consider their relevance to key groups (e.g., healthcare providers, users, and policy makers). | 9-14 |
| Limitations | 25 | Discuss limitations at study and outcome level (e.g., risk of bias), and at review-level (e.g., incomplete retrieval of identified research, reporting bias). | 14、15 |
| Conclusions | 26 | Provide a general interpretation of the results in the context of other evidence, and implications for future research. | 15 |
| **FUNDING** | | | |
| Funding | 27 | Describe sources of funding for the systematic review and other support (e.g., supply of data); role of funders for the systematic review. | 16 |

**PRISMA Checklist** *From:* Moher D, Liberati A, Tetzlaff J, Altman DG, The PRISMA Group (2009). Preferred Reporting Items for Systematic Reviews and Meta-Analyses: The PRISMA Statement. PLoS Med 6(6): e1000097. doi:10.1371/journal.pmed1000097

**Supplement Table 2. Detailed search strategy.**

| # | Searches |
| --- | --- |
| 1 | Hydrogel*[Title/Abstract] |
| 2 | In Situ Hydrogel*[Title/Abstract] |
| 3 | Hydrogel, In Situ*[Title/Abstract] |
| 4 | Patterned Hydrogel*[Title/Abstract] |
| 5 | Hydrogel, Patterned*[Title/Abstract] |
| 6 | 1or 2 or 3 or 4 or 5 |
| 7 | Myocardial Infarction*[Title/Abstract] |
| 8 | Inferior Wall Myocardial Infarction*[Title/Abstract] |
| 9 | Anterior Wall Myocardial Infarction*[Title/Abstract] |
| 10 | Non-ST Elevated Myocardial Infarction*[Title/Abstract] |
| 11 | ST Elevation Myocardial Infarction*[Title/Abstract] |
| 12 | Infarction, Myocardial*[Title/Abstract] |
| 13 | Cardiovascular Stroke*[Title/Abstract] |
| 14 | Stroke, Cardiovascular*[Title/Abstract] |
| 15 | Myocardial Infarct*[Title/Abstract] |
| 16 | Infarct, Myocardial*[Title/Abstract] |
| 17 | Heart Attack*[Title/Abstract] |
| 18 | 7 or 8 or 9 or 10 or 11 or 12 or13 or 14 or 15 or 16 or 17 |
| 19 | 6 and 18 |

**Supplement Table 3. Study inclusion and exclusion criteria.**

| **Inclusion Criteria** | |
| --- | --- |
| #1 | Must be a preclinical experiment conducted in a constructed animal model |
| #2 | The treatment period must be greater than one week |
| #3 | Must be injectable hydrogel and combination therapy for cardiac repair |
| #4 | There must be a separate control group for injectable hydrogel therapy |
| #5 | Myocardial infarction disease model must be constructed by ligating the left coronary artery method |
| #6 | Experimental data for Ejection Fraction or Fractional Shortening must be present for the experimental results |
| #7 | Sufficient experimental data must be provided to calculate the magnitude of the effect |
| **Exclusion Criteria** | |
| #1 | Human clinical trial |
| #2 | Non-injectable hydrogel |
| #3 | Results do not include experimental data for left ventricular ejection fraction or short-axis rate |
| #4 | Control group setting is not reasonable |
| #5 | Immunogenic reactions in animal models during the experiment |
| #6 | Insufficient experimental data to support data analysis |
| #7 | Reviews, editorials, case studies and conference abstracts |

**Supplement Table 4. Study quality assessment using the Heyland methodological quality. score.**

|  | **Design** | | | | **Sample** | | | **Intervention** | | | | | | **MQS**  **(n/18)** |  |
| --- | --- | --- | --- | --- | --- | --- | --- | --- | --- | --- | --- | --- | --- | --- | --- |
| **Reference (Study,year)** | **Randomization(n/2)** | **Blinding**  **(n/2)** | | **Analysis**  **(n/2)** | **Selection**  **(n/1)** | **Comparability(n/2)** | **Follow-up(n/2)** | **Protoco1**  **(n/2)** | **Co-interventions**  **(n/2)** | | **Outcomes**  **(n/2)** | | |  |  |
| Abdul Jalil Rufaihah，2013 | 1 | 1 | 2 | | 2 | 1 | 1 | 1 | 2 | | | 2 | **13** | |  |
| Abdul Jalil Rufaihah，2017 | 1 | 1 | 2 | | 2 | 1 | 1 | 1 | 2 | | | 2 | **13** | |  |
| Adam J. Rocker，2022 | 1 | 1 | 2 | | 2 | 1 | 0 | 1 | 1 | | | 2 | **11** | |  |
| Amanda N. Steele,2018 | 1 | 0 | 2 | | 2 | 0 | 1 | 1 | 2 | | | 2 | **11** | |  |
| Amanda N. Steele,2020 | 1 | 1 | 2 | | 2 | 1 | 0 | 1 | 2 | | | 2 | **12** | |  |
| Andre Chow，2017 | 1 | 0 | 2 | | 2 | 0 | 1 | 1 | 2 | | | 2 | **11** | |  |
| Ann C. Gaffey,2015 | 1 | 0 | 2 | | 2 | 0 | 1 | 1 | 1 | | | 2 | **10** | |  |
| Aparna R. Chakravarti,2018 | 1 | 1 | 2 | | 2 | 1 | 1 | 1 | 2 | | | 2 | **13** | |  |
| Arghya Paul，2014 | 1 | 0 | 2 | | 2 | 0 | 1 | 1 | 2 | | | 2 | **11** | |  |
| Bei Qian，2022 | 1 | 1 | 2 | | 2 | 1 | 1 | 1 | 1 | | | 2 | **12** | |  |
| Bin Xu，2016 | 1 | 1 | 2 | | 2 | 1 | 0 | 1 | 2 | | | 2 | **12** | |  |
| Bjarke Follin，2018 | 1 | 1 | 2 | | 2 | 1 | 1 | 1 | 2 | | | 1 | **12** | |  |
| Bo Fu，2021 | 1 | 0 | 2 | | 2 | 1 | 1 | 1 | 2 | | | 2 | **12** | |  |
| Brendan P. Purcell，2014 | 1 | 1 | 2 | | 2 | 1 | 1 | 1 | 2 | | | 2 | **13** | |  |
| Brendan P. Purcell,2018 | 1 | 1 | 2 | | 2 | 0 | 1 | 1 | 2 | | | 2 | **12** | |  |
| Cagla Eren Cimenci，2021 | 1 | 1 | 2 | | 2 | 1 | 0 | 1 | 2 | 2 | | | **12** | |  |
| Caixia Fan,2019 | 1 | 1 | 2 | | 2 | 1 | 1 | 1 | 2 | 2 | | | **13** | |  |
| Carol W. Chen，2017 | 1 | 0 | 2 | | 2 | 0 | 1 | 1 | 2 | 0 | | | **9** | |  |
| Chan Woo Kim,2020 | 1 | 1 | 2 | | 2 | 1 | 1 | 1 | 2 | 2 | | | **13** | |  |
| Chaoshan Han，2019 | 1 | 0 | 2 | | 2 | 0 | 0 | 1 | 2 | 2 | | | **10** | |  |
| Chien-Hsi Chen,2014 | 1 | 1 | 2 | | 2 | 1 | 1 | 1 | 1 | 2 | | | **12** | |  |
| Chien-Hsi Chen，2016 | 1 | 1 | 2 | | 2 | 1 | 1 | 1 | 1 | 2 | | | **12** | |  |
| Delia Projahn，2014 | 1 | 1 | 2 | | 2 | 0 | 1 | 1 | 2 | 2 | | | **12** | |  |
| Eva Mathieu，2012 | 1 | 0 | 2 | | 2 | 1 | 0 | 1 | 2 | 2 | | | **11** | |  |
| Guohui Xu，2014 | 1 | 1 | 2 | | 2 | 0 | 1 | 1 | 1 | 2 | | | **11** | |  |
| Guoqin Chen,2017 | 1 | 1 | 2 | | 2 | 1 | 1 | 1 | 2 | 2 | | | **13** | |  |
| H. K. Awada,2017 | 1 | 0 | 2 | | 2 | 0 | 1 | 1 | 2 | 2 | | | **11** | |  |
| Haibin Wang，2010 | 1 | 0 | 2 | | 2 | 1 | 1 | 1 | 1 | 2 | | | **11** | |  |
| Haibin Wang，2011 | 1 | 1 | 2 | | 2 | 0 | 1 | 1 | 2 | 2 | | | **12** | |  |
| Haibin Wang，2014 | 1 | 1 | 2 | | 2 | 0 | 1 | 1 | 2 | 1 | | | **11** | |  |
| Hao Ding,2022 | 1 | 1 | 2 | | 2 | 1 | 1 | 1 | 2 | 2 | | | **13** | |  |
| Hekai Li，2018 | 1 | 0 | 2 | | 2 | 1 | 1 | 1 | 2 | 2 | | | **12** | |  |
| Hekai Li，2021 | 1 | 1 | 2 | | 2 | 1 | 1 | 1 | 2 | 2 | | | **13** | |  |
| HongLing Zhu，2017 | 1 | 1 | 2 | | 2 | 1 | 0 | 1 | 2 | 2 | | | **12** | |  |
| Jeffrey E. Cohen, 2014 | 1 | 1 | 2 | | 2 | 0 | 0 | 1 | 2 | 2 | | | **11** | |  |
| Jeffrey E. Cohen，2020 | 1 | 1 | 2 | | 2 | 0 | 1 | 1 | 2 | 1 | | | **11** | |  |
| Jie Ding,2020 | 1 | 1 | 2 | | 2 | 1 | 0 | 1 | 2 | 1 | | | **11** | |  |
| Jin Zhou，2021 | 1 | 1 | 2 | | 2 | 0 | 1 | 1 | 1 | 2 | | | **11** | |  |
| Jingrui Chen，2020 | 1 | 1 | 2 | | 2 | 0 | 1 | 1 | 2 | 2 | | | **12** | |  |
| Jinling Chen，2013 | 1 | 1 | 2 | | 2 | 1 | 1 | 1 | 2 | 2 | | | **13** | |  |
| Jun Wu，2011 | 1 | 1 | 2 | | 2 | 1 | 0 | 1 | 1 | 2 | | | **11** | |  |
| Kashif Khan,2021 | 1 | 1 | 2 | | ` | 0 | 1 | 1 | 2 | 2 | | | **`** | |  |
| Ke Zhu，2022 | 1 | 1 | 2 | | 2 | 1 | 1 | 1 | 1 | 2 | | | **12** | |  |
| Kui Wu，2023 | 1 | 1 | 2 | | 2 | 1 | 0 | 1 | 2 | 2 | | | **12** | |  |
| Leila Montazeri，2020 | 1 | 1 | 2 | | 2 | 1 | 1 | 1 | 2 | 2 | | | **13** | |  |
| Lewis A. Reis,2015 | 1 | 0 | 2 | | 2 | 0 | 1 | 1 | 2 | 2 | | | **11** | |  |
| Long Binh. Vong,2018 | 1 | 0 | 2 | | 2 | 0 | 1 | 1 | 2 | 2 | | | **11** | |  |
| Long Gao，2020 | 1 | 1 | 2 | | 2 | 1 | 1 | 1 | 2 | 2 | | | **13** | |  |
| Maria Chiara Ciuffreda,2018 | 1 | 1 | 2 | | 2 | 1 | 1 | 1 | 1 | 2 | | | **12** | |  |
| MingYao Chang,2016 | 1 | 1 | 2 | | 2 | 0 | 1 | 1 | 1 | 2 | | | **11** | |  |
| Minna H. Chen,2019 | 1 | 0 | 2 | | 2 | 0 | 1 | 1 | 2 | 2 | | | **11** | |  |
| Myeongjin Song，2013 | 1 | 1 | 2 | | 2 | 1 | 1 | 1 | 2 | 2 | | | **13** | |  |
| Quan Qi,2020 | 1 | 1 | 2 | | 2 | 0 | 1 | 1 | 2 | 2 | | | **12** | |  |
| Rui Bao,2017 | 1 | 1 | 2 | | 2 | 1 | 0 | 1 | 1 | 2 | | | **11** | |  |
| Saman Firoozi,2020 | 1 | 1 | 2 | | 2 | 1 | 1 | 1 | 2 | 2 | | | **13** | |  |
| Shilan Shafei，2022 | 1 | 1 | 2 | | 2 | 1 | 1 | 1 | 2 | 2 | | | **13** | |  |
| Shuanghong Lu，2009 | 1 | 1 | 2 | | 2 | 1 | 1 | 1 | 2 | 2 | | | **13** | |  |
| Shuanghong Lu，2010 | 1 | 1 | 2 | | 2 | 1 | 0 | 1 | 2 | 2 | | | **12** | |  |
| Shuoji Zhu，2021 | 1 | 0 | 2 | | 2 | 1 | 1 | 1 | 2 | 2 | | | **12** | |  |
| Shuqin Wang，2022 | 1 | 1 | 2 | | 2 | 0 | 1 | 1 | 2 | 2 | | | **12** | |  |
| Siyuan Bao，2022 | 1 | 1 | 2 | | 2 | 1 | 1 | 1 | 2 | 2 | | | **13** | |  |
| Tao Wang，2009 | 1 | 1 | 2 | | 2 | 0 | 0 | 1 | 2 | 2 | | | **11** | |  |
| Tao Wang，2009 | 1 | 1 | 2 | | 2 | 0 | 1 | 1 | 1 | 2 | | | **11** | |  |
| Thang Duc Vu,2015 | 1 | 1 | 2 | | 2 | 0 | 1 | 1 | 1 | 2 | | | **11** | |  |
| Thomas P. Kraehenbuehl，2010 | 1 | 1 | 2 | | 2 | 0 | 1 | 1 | 2 | 2 | | | **12** | |  |
| Tong Hao,2017 | 1 | 0 | 2 | | 2 | 0 | 1 | 1 | 1 | 2 | | | **10** | |  |
| Wei Wang，2018 | 1 | 1 | 2 | | 2 | 0 | 1 | 1 | 2 | 2 | | | **12** | |  |
| WeiGuo Wan,2013 | 1 | 1 | 2 | | 2 | 0 | 0 | 1 | 2 | 1 | | | **10** | |  |
| WenNing Lu，2009 | 1 | 0 | 2 | | 2 | 1 | 1 | 1 | 2 | 2 | | | **12** | |  |
| Xia Li,2014 | 1 | 1 | 2 | | 2 | 1 | 1 | 1 | 2 | 2 | | | **13** | |  |
| Xiao-Yan Li，2010 | 1 | 1 | 2 | | 2 | 1 | 1 | 1 | 2 | 2 | | | **13** | |  |
| Xinyi Hu，2022 | 1 | 1 | 2 | | 2 | 0 | 1 | 1 | 2 | 2 | | | **12** | |  |
| Yan Li，2021 | 1 | 1 | 2 | | 2 | 1 | 1 | 1 | 2 | 2 | | | **13** | |  |
| Yang Liu，2021 | 1 | 1 | 2 | | 2 | 1 | 0 | 1 | 1 | 2 | | | **11** | |  |
| Yong Wu，2020 | 1 | 1 | 2 | | 2 | 1 | 1 | 1 | 2 | 2 | | | **13** | |  |
| Youhu Chen，2020 | 1 | 1 | 2 | | 2 | 1 | 0 | 1 | 2 | 2 | | | **12** | |  |
| Youming Zhang，2021 | 1 | 0 | 2 | | 2 | 1 | 1 | 1 | 2 | 2 | | | **12** | |  |
| Yu Xia，2015 | 1 | 1 | 2 | | 2 | 0 | 0 | 1 | 2 | 2 | | | **11** | |  |
| Yuanning Lyu，2020 | 1 | 0 | 2 | | 2 | 1 | 1 | 1 | 1 | 2 | | | **11** | |  |
| Yutaka Sakakibara,2002 | 1 | 1 | 2 | | 2 | 1 | 1 | 1 | 2 | 2 | | | **13** | |  |
| Zhi Zheng1,2022 | 1 | 1 | 2 | | 2 | 1 | 1 | 1 | 1 | 2 | | | **12** | |  |
| Zhi Zheng2,2022 | 1 | 1 | 2 | | 2 | 1 | 1 | 0 | 2 | 2 | | | **12** | |  |
| Zhiqiang Liu，2012 | 1 | 1 | 2 | | 2 | 1 | 1 | 1 | 2 | 2 | | | **13** | |  |
| Zhize Yuan，2019 | 1 | 1 | 2 | | 2 | 1 | 1 | 1 | 2 | 2 | | | **13** | |  |

The Heyland MQS assigns a score of 0 or 1 or from 0 to 2 over 9 categories of quality related to study design, sampling procedures, and interventions for a total of 18 points. Randomization was scored 2 points for being randomized with the methods described, 1 point for being randomized without the methods described, or 0 points for not being randomized. Blinding was scored 1 point for being blinded or 0 points for not blinded or not described. Analysis was scored 2 points for being intention-to-treat; all other types of analyses scored 0 points. Sample selection was scored 2 points for being consecutive eligible or 0 points for being preselected or indeterminate. Sample comparability was scored 1 point for being comparable or 0 points for not being comparable at baseline according to either ejection fraction or fractional shortening measurements. Follow-up was scored 1 point for being 100% or 0 points for <100%. Treatment protocol was scored 1 point for being reproducibly described or 0 points for being poorly described. Co-interventions were scored 2 points for being described and equal, 1 point for being described but unequal or indeterminate, or 0 points for not being described. Treatment outcomes were scored 2 points for being described and equal, 1 point for being described but unequal or indeterminate, or 0 points for not being described. MQS = Heyland Methodological Score

**Supplement Table 5.**

A. Ejection Fraction

| Subgroups | No. of Trials | β[95%CI] | Residual I^2^ (%) | P-value |
| --- | --- | --- | --- | --- |
| Dose（#of cells） | 45 | -0.006 [-0.016,0.003] | 75.77% | 0.190 |
| Duration | 67 | -0.111[-0.535,0.314] | 78.91% | 0.605 |
| time to injection  (weeks) | 67 | -0.981[-2.547,0.584] | 78.64% | 0.215 |
| Injection | 11 | -0.999[-2.372,0.375] | 74.11% | 0.134 |

B. Fractional Shortening

| Subgroups | No. of Trials | β[95%CI] | Residual I^2^ (%) | P-value |
| --- | --- | --- | --- | --- |
| Dose（#of cells） | 36 | -0.02 [-0.097,5.863] | 99.69% | 0.628 |
| Duration | 52 | 1.758[-2.347,5.863] | 99.74% | 0.394 |
| time to injection  (weeks) | 52 | 4.749[-2.815,12.312] | 99.74% | 0.213 |
| Injection | 8 | 5.633[1.144.10.123] | 98.04% | 0.022 |

C. End Systolic Diameter

| Subgroups | No. of Trials | β[95%CI] | Residual I^2^ (%) | P-value |
| --- | --- | --- | --- | --- |
| Dose（#of cells） | 20 | 0.143[-0.010,0.039] | 82.94% | 0.240 |
| Duration | 25 | 0.095[-0.971,1.161] | 82.58% | 0.855 |
| time to injection  (weeks) | 25 | -0.702[-3.236,1.832] | 79.17% | 0.572 |
| Injection | 5 | -0.468[-4.637,3.701] | 86.62% | 0.745 |

D. End Diastolic Diameter

| Subgroups | No. of Trials | β[95%CI] | Residual I^2^ (%) | P-value |
| --- | --- | --- | --- | --- |
| Dose（#of cells） | 19 | 0.005[-0.128,0.024] | 89.19% | 0.530 |
| Duration | 25 | 0.219[-0.637,1.074] | 88.05% | 0.602 |
| time to injection  (weeks) | 25 | 0.228[-1.679,2.135] | 88.01% | 0.807 |
| Injection | 5 | -0.781[-1.756,0.194] | 0.00% | 0.084 |

E. End Diastolic Volume

| Subgroups | No. of Trials | β[95%CI] | Residual I^2^ (%) | P-value |
| --- | --- | --- | --- | --- |
| Dose（#of cells） | 8 | 0.049[-0.051,0.148] | 90.38% | 0.276 |
| Duration | 14 | 0.031[-1.280,1.343] | 85.95% | 0.959 |
| time to injection  (weeks) | 14 | 0.234[-6.176,6.644] | 85.14% | 0.938 |

F. End Systolic Volume

| Subgroups | No. of Trials | β[95%CI] | Residual I^2^ (%) | P-value |
| --- | --- | --- | --- | --- |
| Dose（#of cells） | 7 | 0.024[-0.510,0.558] | 96.32% | 0.913 |
| Duration | 13 | 0.687[-2.762,4.136] | 92.79% | 0.670 |
| time to injection  (weeks) | 13 | 0.217[-22.999,23.432] | 92.76% | 0.984 |

G. Infact size

| Subgroups | No. of Trials | β[95%CI] | Residual I^2^ (%) | P-value |
| --- | --- | --- | --- | --- |
| Dose（#of cells） | 28 | 0.009 [--0.009^3^,0.027] | 86.85% | 0.311 |
| Duration | 40 | -0.179 [1.133,0.776] | 88.68% | 0.707 |
| time to injection  (weeks) | 40 | -0.380[-3.183,2.423] | 88.08% | 0.785 |
| Time to injection | 6 | -0.833[-10.709,9.042] | 91.41% | 0.826 |

H. Wall thickness

| Subgroups | No. of Trials | β[95%CI] | | Residual I^2^ (%) | P-value |
| --- | --- | --- | --- | --- | --- |
| Dose（#of cells） | 20 | | -0.002 [-0.769,0.294] | 81.31% | 0.873 |
| Duration | 29 | | -0.237[-0.769,0.294] | 83.17% | 0.368 |
| time to injection  (weeks) | 29 | | -0.059[2.760,2.641] | 83.27% | 0.964 |
| Time to injection | 5 | | 0.621[-2.630,3.873] | 87.32% | 0.586 |

**Continuous a priori subgroup analyses on (A) Ejection Fraction and (B) Fractional Shortening, (C) End Systolic Diameter, (D) End Diastolic Diameter, (E) End Systolic Volume, (F) End Diastolic Volume, (G) Infarct Size, and (H) Wall Thickness in the included studies.**

β is the slope derived from meta-regression analyses and represents the treatment effect of stem cell embedded scaffolds compared to independent injections of cells for primary and secondary outcomes in the included studies. The residual I^2^ value indicates heterogeneity unexplained by the subgroup and is reported as a percent value, where I^2^ ≤ 50% indicated “moderate” heterogeneity, I² ≥ 50% indicated “substantial” heterogeneity, and ≥ 75% indicated “considerable” heterogeneity. P-value significance for heterogeneity was set as P < 0.10.

**Supplement Figure 1.**

Cochrane risk of bias tool to asses Selection Bias, Performance Bias, Detection Bias, Attrition Bias, and Reporting Bias in studies investigating the effects of stem cell-embedded scaffolds on cardiac repair. Authors’ judgments concerning each risk of bias item are presented as percentages across all included studies.

**Supplement Figure 2.**

A. End Systolic Diameter

B. End Diastolic Diameter

C. End Systolic Volume

D. End Diastolic Volume

E. Infarct Size

F. Wall Thickness

**Forest plots of all trials investigating the effect of hydrogel combination therapy on left ventricular (A) End Systolic Diameter, (B) End Diastolic Diameter, (C) End Systolic Volume, (D) End Diastolic Volume, (E) Infarct Size, and (F) Wall Thickness in the included studies.** Pooled effect estimates (diamonds) are shown: one each for trials using hydrogels, patches, microspheres/beads, and their combination (total). Data are expressed as weighted mean differences with 95% CIs, using generic inverse-variance random-effects models. Between-studies heterogeneity was tested by using the Cochran Q statistic (chi-square) at a significance level of P < 0.05. Reference numbers for each study can be found in Table 1 and list of references.

**Supplement Figure 3.**

A. EF

B. FS

C. End Systolic Diameter

D. End Diastolic Diameter

E. End Systolic Volume

F. Infarct Size

G. Wall Thickness

**Forest plots of all trials investigating the effect of hydrogel combination multitherapy on left ventricular (A) EF, (B) FS, (C) End Systolic Diameter, (D)** **End Diastolic Diameter, (E) End Systolic Volume, (F) Infarct Size, and (G) Wall Thickness in the included studies.** Pooled effect estimates (diamonds) are shown: one each for trials using hydrogels, patches, microspheres/beads, and their combination (total). Data are expressed as weighted mean differences with 95% CIs, using generic inverse-variance random-effects models. Between-studies heterogeneity was tested by using the Cochran Q statistic (chi-square) at a significance level of P < 0.05. Reference numbers for each study can be found in Table 1 and list of references.

**Supplement Figure 4.**

A.EF

B.FS

C. End Systolic Diameter

D. End Diastolic Diameter

E. End Systolic Volume

F. Infarct Size

G. Wall Thickness

**Sensitivity analysis.** A.EF,B.FS,C. End Systolic Diameter, D. End Diastolic Diameter, E. End Systolic Volume, F. Infarct Size and G. Wall Thickness.

**Supplement Figure 5.**

**Meta-regression analysis of variables that may impact changes in Fractional Shortening.** Dichotomous a priori subgroup analysis was performed in a trial investigating the effect of injectable hydrogel combination therapy on infarct size. Mean differences in end systolic diameter in the combination regimen treatment group compared to the injectable hydrogel-only treatment group were grouped by

**A** hydrogel type, **B** combination therapy, **C** sex, **D** small animal model, **E** time of treatment, F Duration, G MQS.

**Supplement Figure 6.**

**Meta-regression analysis of variables that may impact changes in LV End Systolic Diameter.** Dichotomous a priori subgroup analysis was performed in a trial investigating the effect of injectable hydrogel combination therapy on infarct size. Mean differences in end systolic diameter in the combination regimen treatment group compared to the injectable hydrogel-only treatment group were grouped by

**A** hydrogel type, **B** combination therapy, **C** sex, **D** small animal model, **E** time of treatment.

**Supplement Figure 7.**

**Meta-regression analysis of variables that may impact changes in LV End Diastolic Diameter.** Dichotomous a priori subgroup analysis was performed in a trial investigating the effect of injectable hydrogel combination therapy on infarct size. Mean differences in end diastolic diameter in the combination regimen treatment group compared to the injectable hydrogel-only treatment group were grouped by

**A** hydrogel type, **B** combination therapy, **C** sex, **D** small animal model, **E** time of treatment.

**Supplement Figure 8.**

**Meta-regression analysis of variables that may impact changes in LV End Systolic Volume.** Dichotomous a priori subgroup analysis was performed in a trial investigating the effect of injectable hydrogel combination therapy on infarct size. Mean differences in end systolic volume in the combination regimen treatment group compared to the injectable hydrogel-only treatment group were grouped by

**A** hydrogel type, **B** combination therapy, **C** sex, **D** small animal model, **E** time of treatment, **F** duration.

**Supplement Figure 9.**

**Meta-regression analysis of variables that may impact changes in LV End Diastolic Volume.** Dichotomous a priori subgroup analysis was performed in a trial investigating the effect of injectable hydrogel combination therapy on infarct size. Mean differences in end diastolic volume in the combination regimen treatment group compared to the injectable hydrogel-only treatment group were grouped by

**A** hydrogel type, **B** combination therapy, **C** sex, **D** time of treatment, **E** duration.

**Supplement Figure 10.**

**Meta-regression analysis of variables that may impact changes in Infarct Size.** Dichotomous a priori subgroup analysis was performed in a trial investigating the effect of injectable hydrogel combination therapy on infarct size. Mean differences in wall thickness in the combination regimen treatment group compared to the injectable hydrogel-only treatment group were grouped by

**A** hydrogel type, **B** combination therapy, **C** sex, **D** small animal model, **E** time of treatment, **F** duration, **G** MQS, **H** animal model.

**Supplement Figure 11.**

**Meta-regression analysis of variables that may impact changes in Wall Thickness.** Dichotomous a priori subgroup analysis was performed in a trial investigating the effect of injectable hydrogel combination therapy on wall thickness. Mean differences in wall thickness in the combination regimen treatment group compared to the injectable hydrogel-only treatment group were grouped by

**A** hydrogel type, **B** combination therapy, **C** sex, **D** small animal model, **E** time of treatment, **F** duration, **G** animal model.

**Supplement Figure 12.**

**Funnel plot for the effect of Injectable hydrogel combination therapy on (A) End Systolic Diameter, (B) End Diastolic Diameter, (C) End Systolic Volume, (D) End Diastolic Volume, (E) Infarct Size, and (F) Wall Thickness**

**Supplement Figure 13.**

**Funnel plot for the effect of Injectable hydrogel combination therapy on Ejection Fraction in Non-mouse small animal models.**
